# RaPID-Query for fast identity by descent search and genealogical analysis

**DOI:** 10.1093/bioinformatics/btad312

**Published:** 2023-05-11

**Authors:** Yuan Wei, Ardalan Naseri, Degui Zhi, Shaojie Zhang

**Affiliations:** Department of Computer Science, University of Central Florida, Orlando, FL 32816, United States; School of Biomedical Informatics, University of Texas Health Science Center at Houston, Houston, TX 77030, United States; School of Biomedical Informatics, University of Texas Health Science Center at Houston, Houston, TX 77030, United States; Department of Computer Science, University of Central Florida, Orlando, FL 32816, United States

## Abstract

**Motivation:**

Due to the rapid growth of the genetic database size, genealogical search, a process of inferring familial relatedness by identifying DNA matches, has become a viable approach to help individuals finding missing family members or law enforcement agencies locating suspects. A fast and accurate method is needed to search an out-of-database individual against millions of individuals. Most existing approaches only offer all-versus-all within panel match. Some prototype algorithms offer one-versus-all query from out-of-panel individual, but they do not tolerate errors.

**Results:**

A new method, random projection-based identity-by-descent (IBD) detection (RaPID) query, is introduced to make fast genealogical search possible. RaPID-Query identifies IBD segments between a query haplotype and a panel of haplotypes. By integrating matches over multiple PBWT indexes, RaPID-Query manages to locate IBD segments quickly with a given cutoff length while allowing mismatched sites. A single query against all UK biobank autosomal chromosomes was completed within 2.76 seconds on average, with the minimum length 7 cM and 700 markers. RaPID-Query achieved a 0.016 false negative rate and a 0.012 false positive rate simultaneously on a chromosome 20 sequencing panel having 86 265 sites. This is comparable to the state-of-the-art IBD detection method TPBWT(out-of-sample) and Hap-IBD. The high-quality IBD segments yielded by RaPID-Query were able to distinguish up to fourth degree of the familial relatedness for a given individual pair, and the area under the receiver operating characteristic curve values are at least 97.28%.

**Availability and implementation:**

The RaPID-Query program is available at https://github.com/ucfcbb/RaPID-Query.

## 1 Introduction

With the popularity of direct-to-consumer (DTC) genotyping services, genetic databases are growing to tens of millions of individuals (https://thednageek.com/dna-tests/). Thus, using single nucleotide polymorphisms (SNPs) data from autosomal chromosomes to perform genealogical search has become feasible for familial relationship inference. DTC companies provide such a genealogy service to predict the degree of relationships using accumulated shared identity-by-descent (IBD) segments between each customer and the genetic database of collected genetic data of their customers. In early days, IBD segments were identified from a pairwise comparison-based detection algorithm (Henn et al. 2012). Currently, efficient IBD detection methods have emerged and are available for relatedness inference, such as PBWT (Durbin 2014), PBWT-Query (Naseri et al. 2019a), RaPID (Naseri et al. 2019b), Hap-IBD (Zhou et al. 2020a), FastSMC (Nait Saada et al. 2020), TPBWT (Freyman et al. 2021), d-PBWT (Sanaullah et al. 2021), and iLASH (Shemirani et al. 2021). Though these latest methods show the potential power of IBD driven approach in relatedness studies (Sticca et al. 2021), most of them are not able to perform query search, a process that takes an individual’s genetic data as the query and searches the population genetic database for IBD segments. All-versus-all IBD detection methods become less practical due to the large and continuously growing size of genetic databases, as they take times to go over tens of millions of individuals for each new individual query. Thus, one-versus-all IBD detection methods are more suitable for the large database circumstance, by saving runtimes from examining all individuals in the database repeatedly.

For real-time query search, Freyman et al. proposed a batch-based method named TPBWT(out-of-sample) (Freyman et al. 2021). TPBWT(out-of-sample) pre-processes the data panel by compressing it and storing it in a binary format on the disk. For any new individual queries, it compresses them the same way as it does for the panel and loaded the compressed panel from the file and appends the compressed queries to it. Then, TPBWT(out-of-sample) performs the same IBD detection method as TPBWT does, except only outputting IBDs between the panel and the queries. Naseri et al. and Sanaullah et al. proposed index-based query approaches (Naseri et al. 2019a; Sanaullah et al. 2021) that are promising. Both approaches convert data panel into Positional Burrows–Wheeler transform (PBWT) (Durbin 2014) format and host it in memory. Then, they run their efficient haplotype matching algorithms derived from Durbin (2014) to make IBD calls. Naseri et al. proposed PBWT-Query, a long match-based query algorithm, i.e. able to effectively find IBD segments in a given panel for a given query. It is a more practical formulation for genealogical search than Durbin’s set-maximal match-based query algorithm, as it allows specifying the minimal length of the match. Naseri et al. also proposed L-PBWT-Query which uses an additional data structure LEAP array to achieve run-time efficiency (Naseri et al. 2019a). Sanaullah et al. further improved the PBWT-Query algorithm by proposing the triple sweep long match query and the single sweep long match query algorithms. Comparing to Naseri et al.’s algorithm, Sanaullah et al.’s algorithms efficiently reduce the memory usage while keeping a fast query time. The triple sweep long match query algorithm theoretically achieves a linear runtime complexity. For a chromosome 21 panel with 974 618 haplotypes and 9793 sites, the average query time of matches with at least one thousand sites for the single sweep long match query algorithm is 2.13 ms for PBWT implementation, and 3.55 ms for d-PBWT implementation (Sanaullah et al. 2021). In addition, Sanaullah et al. proposed a linear panel update algorithms (i.e. insertion and deletion) to dynamically update the data panel in PBWT format in memory, when it is needed to add a new individual or remove an existing individual from the panel.

However, Naseri et al.’s and Sanaullah et al.’s query search algorithms are still not practical for genealogical search in real databases because they do not allow any mismatches when performing IBD segment detection. IBD segments may have mismatched sites due to mutation, gene conversion, or genotyping error. The exact IBD segment matching result from Naseri et al.’s or Sanaullah et al.’s algorithm may underestimate the length of the real IBD segment, which may impact the degree of relatedness inference. Thus, a new efficient haplotype query method tolerating mismatches is desired.

Here, we propose a new PBWT-based IBD detection method, random projection-based identity-by-descent (IBD) detection (RaPID) query, referred to as RaPID-Query. To allow mismatches while maintaining efficiency and accuracy, RaPID-Query uses the idea of multiple randomly projected low-resolution PBWT panels introduced in RaPID (Naseri et al. 2019b). In addition, a few algorithmic innovations are introduced. First, we use x-PBWT-Query, an extended PBWT query algorithm, by simplifying Sanaullah et al.’s single sweep long match query algorithm. We eliminate the use of virtual query indicator and the steps of divergence value comparisons each time when initializing a new long match block. We offer a site distance tracking feature which makes the algorithm flexible to IBD target length on both physical and genetic unit of measurement. Second, we come up with a multi-resolution PBWT idea: while merging the results from the multiple low-resolution PBWT scans which gives approximate IBD segments, a high-resolution PBWT is run to refine the result with additional segmental reconstruction. A new merging method is proposed, tailored to queries, requiring no intermediate output to hard disk in contrast to RaPID. Sanaullah et al.’s panel update algorithms are still viable to RaPID-Query since updating the panel does not have any matter of site mismatching issue. Thus, RaPID-Query provides a promising method for fast and efficient query search. We compare the performance of RaPID-Query with TPBWT(out-of-sample) as well as the state-of-the-art all-versus-all IBD detection method Hap-IBD (Zhou et al. 2020a). We investigate the feasibility of the query time on large cohort dataset for RaPID-Query. We also perform the analysis of individual relatedness degree separation on IBD segments detected by RaPID-Query and compare the result with x-PBWT-Query.

## 2 Materials and methods

The RaPID-Query method uses a new long match query algorithm, named x-PBWT-Query algorithm [i.e. extended PBWT query algorithm, by simplifying Sanaullah et al.’s single sweep long match query algorithm (Sanaullah et al. 2021)] to detect the IBD segment, with the random projection trait to tolerate mismatching sites in IBD segment. The resulting IBD segments from the query are refined by running long match query algorithm on the full resolution panel. The random projection trait is inherited from RaPID (Naseri et al. 2019b). A new merging algorithm tailored to querying is proposed, which requires no additional disk space as there are no intermediate files output to the disk. Compared to PBWT query algorithms (Naseri et al. 2019a; Sanaullah et al. 2021), the IBD segments from RaPID-Query method are with high quality and allowing mismatch sites for the query search.

### 2.1 x-PBWT-Query

The x-PBWT-Query algorithm is a simplified version of Sanaullah et al.’s single sweep long match query algorithm (Sanaullah et al. 2021). The algorithm takes a query and a panel with *n* number of sites as the input, as well as the minimum cutoff length of the IBD segment *L* (where L∈[1,n]). It outputs the IBD segments found between the query individual and individual in the panel [i.e. it reports a tuple (individual_id_in_panel, IBD_start_position, IBD_end_position) for the query_individual_id]. The algorithm assumes PBWT panels (i.e. prefix array *p*, divergence array *d*, and block indicator related arrays *u* and *v*) (Durbin 2014) are pre-computed and accessible. The x-PBWT-Query algorithm pseudocode can be found in Supplementary Algorithm S1, and the detail of the getBlockIndicator(⋅) function to update the match block [f,g) indicators *f* (or *g*) in Supplementary Algorithm S1 is described in Supplementary Algorithm S2, which covers both regular and boundary cases.

Sanaullah et al.’s single sweep long match query algorithm (Sanaullah et al. 2021) assumes the query haplotype is virtually inserted into the panel. A virtual query indicator is used to track the query haplotype location in the panel. The algorithm adopts the set-maximal match mechanism, first introduced by Durbin (2014), to find the long match block. This is similar to the behavior of Naseri et al.’s PBWT-Query algorithm (Naseri et al. 2019a). If the length of the start position *e* of the set-maximal match to the current scanned site position *k* is at least the cutoff long match length *L*, a match block is formed. The newly formed match block has one haplotype only, whose location depends on the values of block indicator arrays *u* and *v*, as well as the virtual query indicator. Then, the algorithm uses the neighbor haplotypes’ divergence values to expand the match block in both directions. If such divergence value shows the length of the match between the haplotype in the match block edge and the neighbor haplotype outside of the match block edge is at least k+1−L, the match block is expanded. Finally, the algorithm reports matches if the matches in the match block that have the opposite site value to that of its neighbor’s haplotype (i.e. a break) in the next-to-be scanned site position.

**Figure 1.**
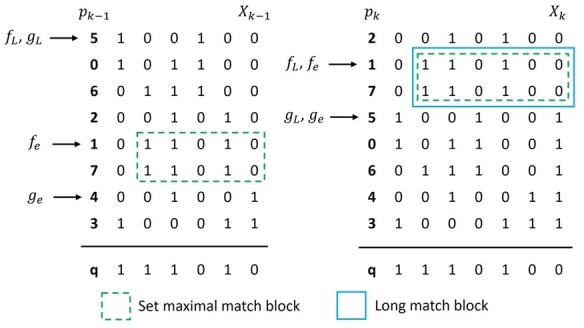
x-PBWT-Query long match block initialization example (L=6): it starts with set maximal match block $\left[f_{e},g_{e}\right)$ with match length k−1−(k−5)+1=5, resulted from Durbin’s set maximal match query algorithm on site k−1; then on site *k*, the long match block $\left[f_{L},g_{L}\right)$ is initialized as block $\left[f_{e},g_{e}\right)$ since the match length threshold is satisfied (i.e. k−(k−5)+1=L).

The proposed x-PBWT-Query algorithm simplifies the Sanaullah et al.’s single sweep long match query algorithm when forming the match block. Firstly, it does not use the concept of virtual insertion thus tracking the virtual query indicator is no longer needed. Secondly, it eliminates the redundant steps of evaluating the divergence values of the haplotypes if they are already in the set-maximal match block. In x-PBWT-Query algorithm, the step of updating the value of the virtual query indicator in each site is not needed. The long match block is initially formed to include all haplotypes in the set-maximal match block when the constraint “e=k+1−L” is satisfied. Figure 1 is an example of the long match block initialization process when L=6. Compared to the procedure of initializing match block in Sanaullah et al.’s single sweep long match query algorithm, where the match block starts with one haplotype, the x-PBWT-Query algorithm’s match block starts with all haplotypes in the set-maximal match block. This approach simplifies the match block build-up process by avoiding re-evaluating the divergence values of the haplotypes already in the set-maximal match block during the step of expanding the match block. Though theoretically the time complexity stays the same, it provides a simpler way of implementation in practical applications.

**Figure 2.**
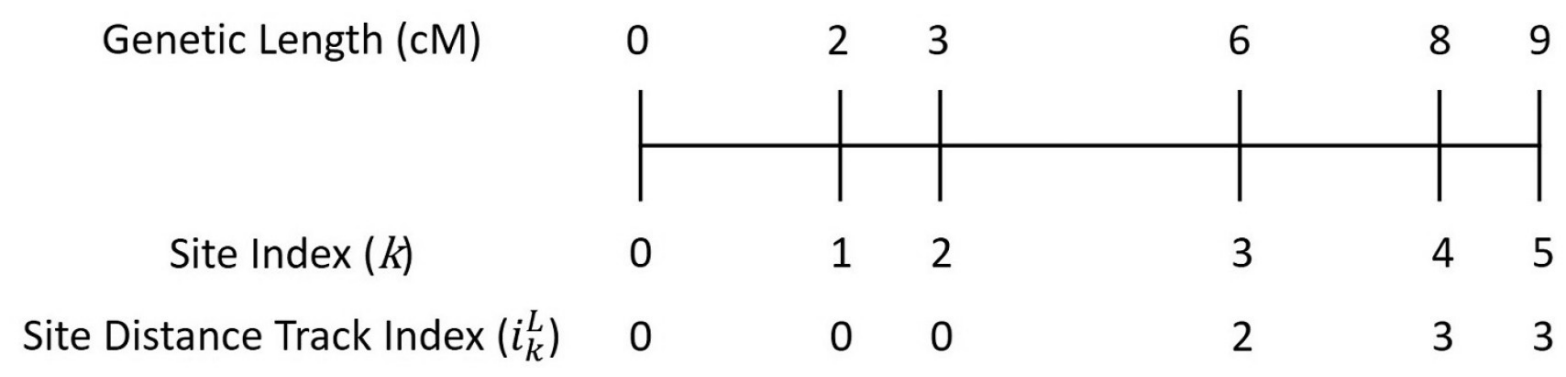
x-PBWT-Query site distance track index example (L=2): initially $i_{k}^{2}=0$; if the genetic distance between site *k* and k−1 is less than the threshold (i.e. L=2), $i_{k}^{2}=i_{k-1}^{2}$ (which is the case when k∈{1,2,5}); otherwise, $i_{k}^{2}=k-1$ (which is the case when k∈{3,4}).

In addition, The x-PBWT-Query algorithm offers a new feature: site distance tracking. This feature makes x-PBWT-Query algorithm feasible when the cutoff long match length *L* is in either physical or genetic unit of measurement. The algorithm tracks site index *i* who is *L* away from the currently scanned site *k* while maintaining the same complexity of the algorithm. Figure 2 is an example of the updates of the site distance track index *i* in scanned site *k*. Site distance track index $i_{L}^{k}$ indicates the closest site having *L* centiMorgan (cM) distance away from site *k*. The site distance track index *i* is updated as the site scanning goes. Since the site scanning would never go backwards, the maximum number of the update is bounded by the number of sites (i.e. linear operation). When expanding the match block, the site distance track index *i* is marked as the start position of the matches in the match block. In Sanaullah et al.’s single sweep long match query algorithm, the start position of the matches is updated as k+1−L, which requires non-trivial conversion (e.g. an additional data structure holding the mapping information from genetic positions to physical positions is needed) if the given cutoff long match length *L* is in genetic distance format. The site distance track index of x-PBWT-Query algorithm facilitates the usage of the algorithm in real applications. In most real world scenarios, genetic maps are used to measure distance in chromosomes; thus, genetic length is used to represent the cutoff long match length *L*. In such cases, it is very straightforward to have genetic maps directly applied to the distance track variable *i* in x-PBWT-Query algorithm, to be able to query the panel easily using the genetic distance cutoff length.

The time complexity of the proposed x-PBWT-Query algorithm is O(nm) in worst case scenario, where *n* is the number of sites and *m* is the number of haplotypes. In the average case, its time complexity is O(n+|output|), where |output|∈O(nm) is the number of the output IBD segments. This is equivalent to the time complexity of Durbin’s finding all set-maximal matches from a new sequence algorithm (Durbin 2014), Naseri et al.’s PBWT-Query algorithm (Naseri et al. 2019a), and Sanaullah et al.’s single sweep long match query algorithm (Sanaullah et al. 2021). This is because Durbin’s procedure of finding all set-maximal matches from a new sequence, dominates the time complexity since it is part of these algorithms. The empirical evaluation on the average time complexity of this procedure being O(n+|output|) is documented and the result is confirmed (Naseri et al. 2019a). The time complexity of the proposed long match query algorithm independent from the number of haplotypes *m* makes the algorithm scalable to gigantic biobank-sized or even population-scaled panels.

### 2.2 RaPID-Query

**Figure 3.**
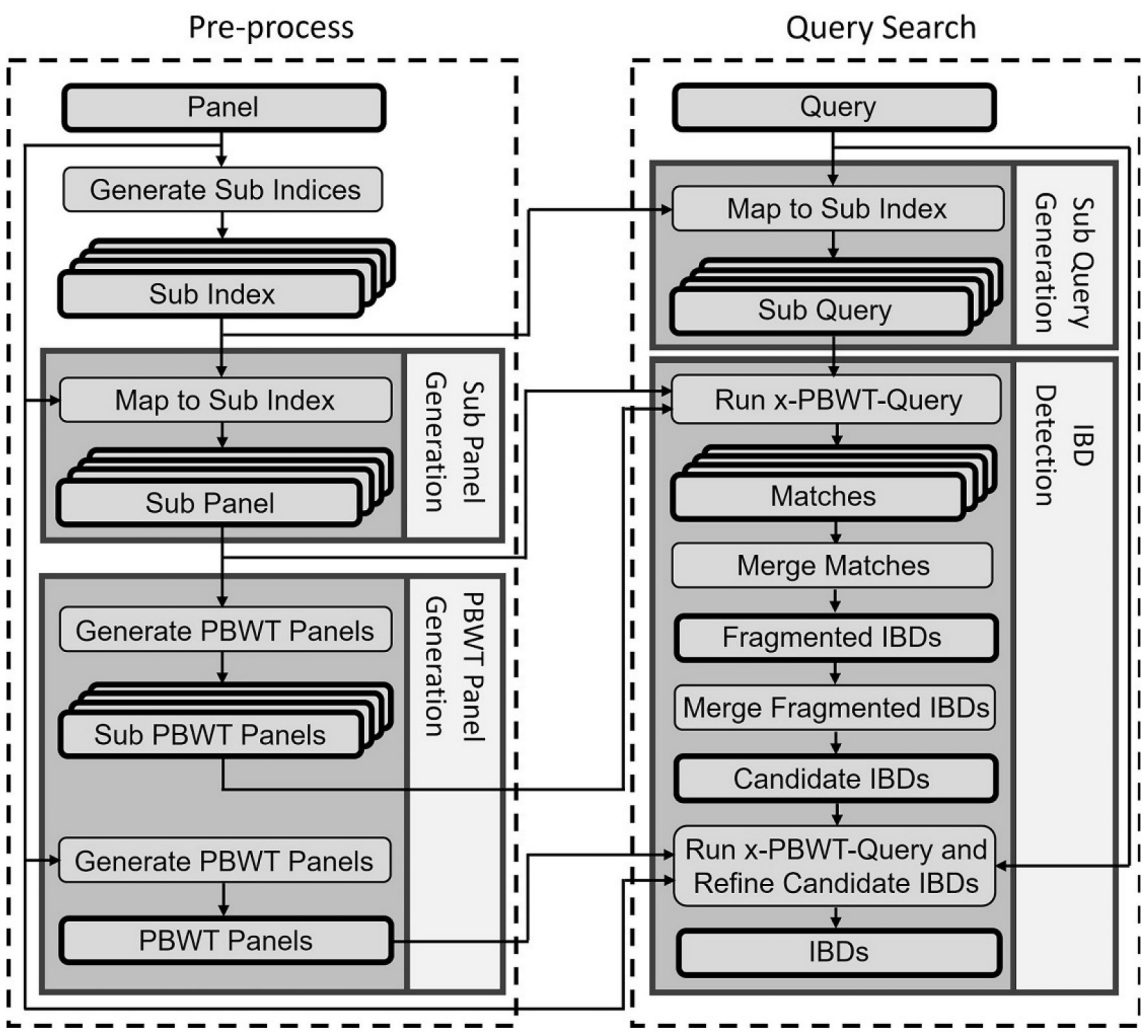
RaPID-Query workflow.

The drawback of PBWT-based long match query algorithm is not allowing mismatch sites when searching for shared IBD segments. It is common that an IBD segment contains a few mismatch sites due to the event of genotyping error, gene conversion, or mutation. To have the proposed long match query algorithm allow mismatch sites in IBD segment, the concept of random projections, originally proposed in RaPID (Naseri et al. 2019b), is brought into the new long match query algorithm and composes the random projection-based IBD detection (RaPID) query method, referred to as RaPID-Query. Similar to RaPID, RaPID-Query divides the panel into windows and for each window a site is sampled randomly on the weight of the site frequency, to form a low resolution panel. RaPID-Query generates multiple low resolution panels and then, the x-PBWT-Query algorithm is run on each low resolution panel to find exact matched segments. The next step is to combine result segments from all the runs. A new IBD segment merging method tailored to querying is proposed to identify canididate IBD segments. Finally, the x-PBWT-Query algorithm is run on the original resolution panel to get the IBD segments in full resolution. The full resolution IBD segments are used to refine the boundaries of low resolution IBD segments, stitching IBDs together if their distance in chromosome region is within the allowed gap $g_{\text{max}}$. Figure 3 shows the entire RaPID-Query workflow. The left section shows the pre-process part: the population panel and associated PBWT panels and their sub panels are calculated in advance. The right section shows the query search part: the associated sub queries are generated based on the provided individual query and search is performed quickly thanks to all the pre-processed panels and sub panels.

#### 2.2.1 Query with random projection

In RaPID (Naseri et al. 2019b), the insight for IBD detection comes from the idea that IBD segments are approximate matches over a long range, which can be mapped to a problem of exact matches of low-resolution sequences with high probability. Specifically, we designed a randomized algorithm that first produces multiple low-resolution PBWTs on random subsets of markers, and then combines the results efficiently using an interval tree data structure. Multiple PBWT runs are needed because that a single run of PBWT on randomly selected markers usually will have low power and accuracy. The tolerance of mismatches is made possible by the projection function at each window that may “collapse” different strings into the same bit. The balance of detection power and false positive is achieved by running a total of *r* rounds of random projections and a “hit” is claimed when *c* out of *r* rounds have a *L*-window PBWT match. For a minimal IBD length $L^{*}$, the window size is $w=L^{*}/L$. Therefore, three parameters for RaPID (and also RaPID-Query) are *r*, *c*, *w* which are determined by statistical calculation (Naseri et al. 2019b).

RaPID-Query follows the same statistical framework. The first part of the method is to produce matches for the query allowing mismatch sites. Assuming long matches with minimum match cutoff length *L* are to be found on a panel with total number of *n* sites and *m* haplotypes for a query haplotype with the same *n* sites. During the pre-process stage, the PBWT panels (i.e. prefix array *p*, divergence array *d*, and block indicator related arrays *u* and *v*) as well as the minor allele frequencies of each site are calculated based on the input site panel. Then, the random projection approach is used to create down-sampled site panels from the original site panel. The site panel is equally divided into $\left\lceil \frac{n}{w}\right\rceil$ sections, where each section’s window size is *w*. The sub index is sampled from the index of the site panel, one site index per section, by using weighted random sampling. The probability of each site index getting selected is proportional to the minor allele frequency of the site in the section. The larger the minor allele frequency the site has, the more chance of such site is getting selected. The sub index is formed with $\left\lceil \frac{n}{w}\right\rceil$ site indices. For each random projection run, one sub index is needed. Thus, there are *r* sub indices generated in the pre-process stage where *r* is the number of random projection runs. Each sub index is correlated to a sub panel. Next, the sub PBWT panels are created for each sub panel and ready for the queries. In the query search stage, *r* sub queries are generated by mapping the input query site panel to the sub indices. Then, x-PBWT-Query algorithm is applied on each sub query and correlated sub panel and sub PBWT panels, to output the detected matches. The detected matches are rescaled from the resolution window size to the original panel and query size. Specifically, a low resolution match $\left(id,[h^{\prime },t^{\prime }]\right)$ found from the low resolution panel with the window size *w*, is rescaled to the original resolution as $\left(id,[wh^{\prime },w(t^{\prime }+1)-1]\right)$ (or (id,[h,t])), where *id* is the haplotype identification number who has the match with the query haplotype, $h^{\prime }$ (or $h=wh^{\prime }$) is the head or start position of the match and $t^{\prime }$ (or $t=w(t^{\prime }+1)-1$) is the tail or end position of the match, inclusively. Supplementary Fig. S1 shows an example of query with random projection runs.

#### 2.2.2 IBD segment identification

**Figure 4.**
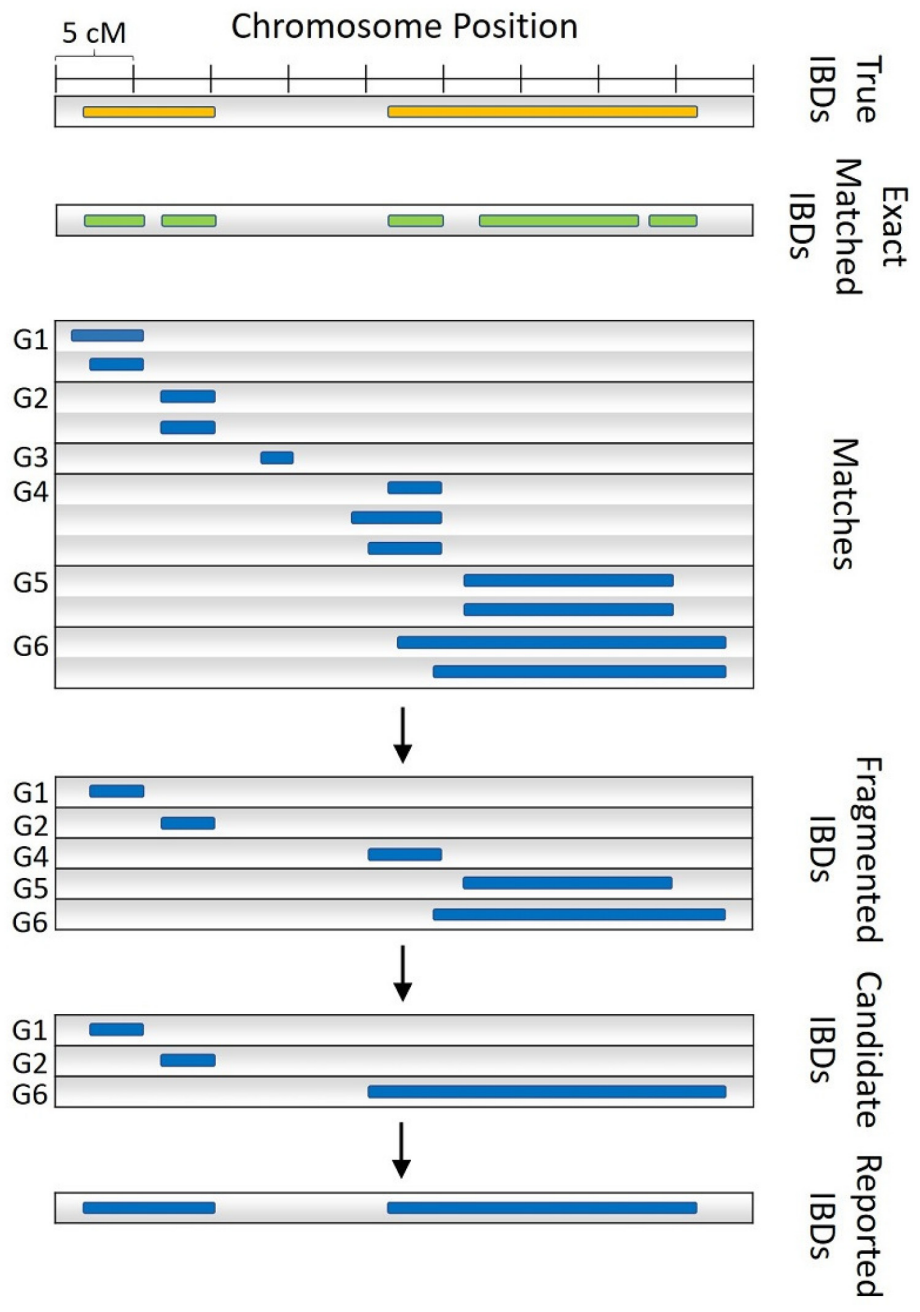
Merge and refine example of a haplotype pair ($c=2,g_{\text{max}}=2\;\text{cM}$). Groups containing no fragmented or candidate IBD segments are omitted.

The second part of the method is to merge and refine the detected matches found from x-PBWT-Query results. Different from RaPID which stores matches from a projection run in an intermediate file in interval tree format on disk and uses counting sort to merge all matches read from multiple files of runs, RaPID-Query uses a query-tailored method with no need of reading or writing any intermediate files or using interval tree or counting sort to identify IBDs. The new merging method is scalable to large numbers of detected IBDs as it eliminates the situation that disk space is run out or runtime is hampered by I/O performance, while it is deterministic so the result is guaranteed to be the same as the one RaPID used. Firstly, the detected matches are grouped by the end position of the match. Secondly, the fragmented IBDs are identified as the match having the *c*th smallest start position in each group, and the other matches are discarded. *c* is the “count” parameter (similar to the one RaPID uses), which is the threshold of keeping the matches from random projection runs. Since matches are aligned by their end positions, in each end position group, it is guaranteed the match having the *c*th smallest start position will completely overlap with at least *c* matches in the group. Thus, such a match is identified as a fragmented IBD. Thirdly, the candidate IBDs are formed by merging the fragmented IBDs, if two fragmented IBDs have overlap. The candidate IBD is formed as the union of the two fragmented IBDs. Lastly, the candidate IBDs are refined by comparing to the exact matched IBDs from the original resolution run using x-PBWT-Query algorithm. The boundaries of candidate IBD who has overlap with the exact matched IBD is extended or trimmed according to the overlapped exact-matched IBD. If the distance between two candidate IBDs is within the maximum gap threshold $g_{\text{max}}$, the IBD is formed from stitching the two candidate IBDs. This is the special case of IBD boundary correction. Figure 4 is an IBD identification example of a haplotype pair with c=2 and $g_{\text{max}}=2\;\text{cM}$. It shows there are six groups of detected matches (i.e. G1–G6) after grouping the end position of the match. Then, five matches having the second smallest start position in each group, are identified as the fragmented IBDs (i.e. G1–G2, G4–G6). The match in G3 is discarded as there is no match having the second smallest start position existing in the group. Then, the fragmented IBDs are merged if they have overlap across the groups. In the example, three candidate IBDs are formed: one from group G1, one from group G2, and the other one from the union of three fragmented IBDs from group G4-G6 since they have overlaps. Finally, the three candidate IBDs are refined into two IBD segments: the candidate IBDs from group G1 and G2 are stitched together, as the gap between them in chromosome region is less than 2 cM; the candidate IBD from the group G6 is trimmed on both ends, to be aligned with the boundaries of the overlapped exact matched IBD segment.

The first step of IBD segment identification is to group the end positions of the detected matches (id,[h,t]) from the random projection runs, using an array of hash tables $B_{a}$. See Supplementary Algorithm S3. If $C_{\text{low}}$ is a set containing all matches from the random projection runs, and *l* is the length of the longest match found at site *t*, the time complexity is $O(\text{max}(\frac{l}{w}n,|C_{\text{low}}|))$, and the space complexity is $O(n+|C_{\text{low}}|)$. The second step is to identify the fragmented IBDs from the detected matches by filtering out the false positive matches. This step only requires a simple traversal on $B_{a}$. See Supplementary Algorithm S4. The time complexity is $O(|C_{\text{low}}|)$, and the space complexity is $O(n+|C_{\text{low}}|)$. The third step is to form the candidate IBDs by merging overlapped fragmented IBDs in $B_{a}$. Supplementary Algorithm S5 shows how fragmented IBDs are evaluated in the order of their ending positions, and are merged or appended if any overlapped segments are found. The time complexity is $O(\text{max}(\frac{l}{w}n,|C_{\text{low}}|))$, and the space complexity is $O(n+|C_{\text{low}}|)$. In the last step, the IBDs are called from the refinement of the candidate IBDs with the guidance of the exact matched IBDs from the run on full resolution panel. To efficiently refine the candidate IBDs, a hash table data structure $B_{m}$ is used to store and refine the candidate IBDs originally found from random projection runs in low resolution panels. Supplementary Algorithm S6 shows the conversion of the candidate IBD form from $B_{a}$ to $B_{m}$ with a sorted order of the candidate IBD locations in a linear fashion. Then, a refine-on-the-fly approach is used: while running the long match query on full resolution panel, if an exact matched IBD is found, and if it has overlap with a candidate IBD, it is used to refine such candidate IBD; otherwise it is discarded. The start and end boundary of the calling IBD lines up with the exact matched IBD found from the run on full resolution panel, which has an overlapping segment with the candidate IBD found from runs on low resolution panels. In addition, the algorithm is allowed to have a gap $g_{\text{max}}$ in a calling IBD. Similar to the maximum gap length idea that Hap-IBD uses (Zhou et al. 2020a), such a gap is considered as a genotyping error, mutation, or gene conversion. This is to rectify the small gap on the IBD by stitching two candidate IBDs whose distance is within the gap distance $g_{\text{max}}$ on a chromosome region, when such a gap is not picked up by random projection runs. Supplementary Fig. S2 shows using such gap parameter improves the quality of called IBDs. The length of the IBD to be reported needs to be at least *L* after the refinement. See Supplementary Algorithm S7. Following the same notation, if $C_{\text{full}}$ denotes a set containing all exact matched IBDs found in full or original resolution panel, the time complexity of IBD refinement is $O(\text{max}(n,|C_{\text{low}}|+|C_{\text{full}}|))$, and the space complexity is $O(n+|C_{\text{low}}|)$.

## 3 Results

To test out the performance of RaPID-Query method, sets of experiments are performed on simulated data and real-world data. The experiments on the simulated dataset show the feasibility of IBD segment detection using RaPID-Query method. The experiments on the real-world dataset prove the practicability of using RaPID-Query method to perform IBD segment-based genealogical searches. The machine used for the experiments has Intel Xeon Gold 5215 2.50 GHz processor with 3 terabytes of RAM.

### 3.1 Dataset

A 2000-haplotype Whole Genome Sequencing (WGS) dataset was simulated to test the performance of RaPID-Query method. The simulation used the out-of-Africa demographic model (Gutenkunst et al. 2009) via msprime v1.0.1 (Kelleher et al. 2016; Baumdicker et al. 2022) with the parameters provided by stdpopsim library (Adrion et al. 2020), using Discrete Time Wright Fisher model (Nelson et al. 2020). The chromosome 20 genetic map in GRCh37 coordinates [from HapMap Phase II project (The International HapMap Consortium. 2007)] was used as the recombination map. The mutation rate was set to 1.38e-08 as it was the same constant rate in simulated dataset tested by the state-of-the-art all-versus-all IBD detection method: Hap-IBD (Zhou et al. 2020a). This mutation rate also falls within the most recent estimated mutation rate 95% confidence interval: [1.02e–8, 1.56e–8] (Tian et al. 2019). The simulated dataset consists 1000 individuals, sampled from the European population. The sites with multi-allelic values or minor allele frequency less than or equal to 1% were filtered out from the original dataset, yielding total 86 265 bi-allelic sites. A typical 0.04% genotyping error rate was added, as observed from TOPMed WGS dataset (Taliun et al. 2021). In addition, a 0.16% phasing error was added using SHAPEIT4 (Delaneau et al. 2019) and verified using VCFtools (Danecek et al. 2011). The ground truth of IBD segment was identified as the contiguous segment where the two individuals have the same most recent common ancestor (MRCA) in the simulated coalescent trees. By adopting the same efficient process as Browning and Browning (2013) did, the trees were sampled per 5000 genome position and the minimum genetic length of a true IBD segment calling is 1 cM.

To show the detected IBD segments from RaPID-Query are robust for genealogical search, the UK biobank SNP-array genotyping dataset (Bycroft et al. 2018) was used as the real-world testbed. All autosomal chromosomes containing 487 409 individuals with 658 719 sites were used to show that the IBD segments have the power of separating different degrees of relationships. The UK biobank dataset contains the ground truth of the relatedness of individuals up to third degree, measured as part of the UK biobank study and presented in the form of the kinship coefficient. The estimation of such kinship coefficient is generated by the kinship coefficient, Kinship-based INference for Genome-wide association studies (KING) software (Manichaikul et al. 2010). The actual degree of relatedness is determined by the ranges where the kinship coefficient resides in: first degree: (0.177, 0.354], second degree: (0.0884, 0.177], and third degree: (0.0442, 0.0884] (Manichaikul et al. 2010).

To demonstrate the robustness of distinguishing the different degrees of relatedness of individuals up to fourth degree using the detected IBD segments from RaPID-Query, 22 chromosomes were simulated. The simulated dataset was created by an in-house forward simulation program mimicing the UK biobank SNP-array genotyping dataset. The genetic map from deCODE (Halldorsson et al. 2019) was used and the cross-overs for each chromosome were calculated using Poisson distribution ($\lambda =L_{t}/50$), where $L_{t}$ is the chromosome length in cM. The mating patterns were designed according to Speed and Balding’s (2015) definition. At each generation up to 500 non-overlapping couples were selected and the chance that a female may have a child with a different male individual from the same generation was set to 0.2. 1000 unrelated individuals from the UK Biobank were randomly selected and the population size at each generation was set to 1000. The genetic data of the last four generations comprised of 4000 individuals including their relationships were extracted. To make the simulated dataset more realistic, a typical 0.13% genotyping error rate was added, as observed from UK biobank SNP-array dataset (Bycroft et al. 2018).

### 3.2 Power and accuracy

The power and accuracy of RaPID-Query is demonstrated by two measurements: false negative rate and false positive rate. They are calculated based on matches: cumulative number of sites overlapped between true IBDs and reported IBDs. In this set of experiments, RaPID-Query with different sets of refinement parameters, indicating the cutoff genetic lengths of exact match IBDs run in original resolution panel, were examined against the x-PBWT-Query method. Generally, the recommended refinement value is 0.5 cM for WGS and 1.0 cM for SNP-array dataset, due to the marker density. The other parameters were inherited from RaPID (Naseri et al. 2019b) and the values were set on an empirical basis. TPBWT (Freyman et al. 2021) was also included in the experiments, which offers a one-versus-all batch-based approach: TPBWT(out-of-sample). Besides RaPID-Query, it is the only known one-versus-all method allowing mismatches. PBWT-Query and L-PBWT-Query were not included because they have the exact same result as x-PBWT-Query since all of them are exact-match-based algorithms, and it takes considerable computational resources for them to run. The results of all 2000 haplotype queries were merged and averaged, in order to compare the results to the non-query-based all-versus-all state-of-the-art method Hap-IBD (Zhou et al. 2020a). The objective of including an all-versus-all method in the experiments is not to provide a comprehensive comparison with them, but to show that one-versus-all methods are able to produce IBD segments as high-quality as all-versus-all methods do. Hap-IBD was selected because it is one of the newest all-versus-all IBD detection methods and it outperforms a few other IBD detection methods published in previous years (Zhou et al. 2020a). We chose the recommended parameters based on an extensive benchmarking of all-versus-all IBD detection methods, including TPBWT and Hap-IBD, conducted on both WGS and SNP-array datasets (Tang et al. 2022), for our performance analysis (see Supplementary Table S1). In general, RaPID-Query is the only method able to achieve low false negative and positive rate simultaneously on WGS and SNP-array datasets.

#### 3.2.1 False negative rate

The measurement of false negative rate is defined as the average proportion of true IBD segments not having been covered by reported IBD segments, i.e. the sum of proportions of true IBD segments not overlapped with reported IBD segments over the number of true IBD segments: where *IT* is true IBD set, *it* is true IBD, *st* is site of true IBD, *IR* is reported IBD set, *ir* is reported IBD, and *si* is the sampled individual ID.


(1) $$\begin{array}{c} \frac{1}{\left|IT\right|}\sum_{it\in IT}\frac{1}{\left|it\right|}\sum_{st\in it}\left[st\notin \{ir\in IR\},\;\text{where}\;ir.si=it.si\right], \end{array}$$

**Figure 5.**
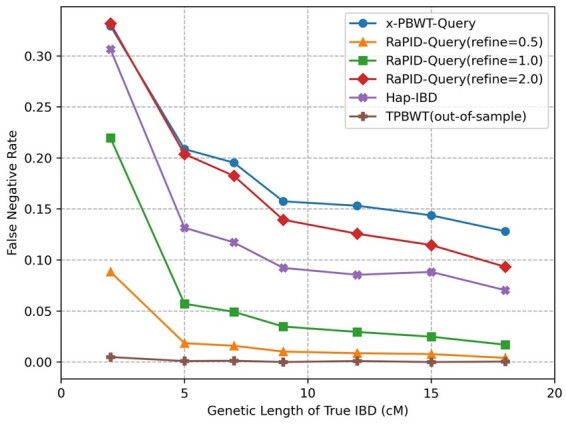
False negative rates. True IBD segments with length ≥2 cM were assigned into bins of 2–5, 5–7, 7–9, 9–12, 12–15, 15–18, and ≥18 cM according to their genetic length. The false negative rate is the proportion of true IBD segments in a bin that are not covered by any reported IBD segment ≥2 cM.

**Figure 6.**
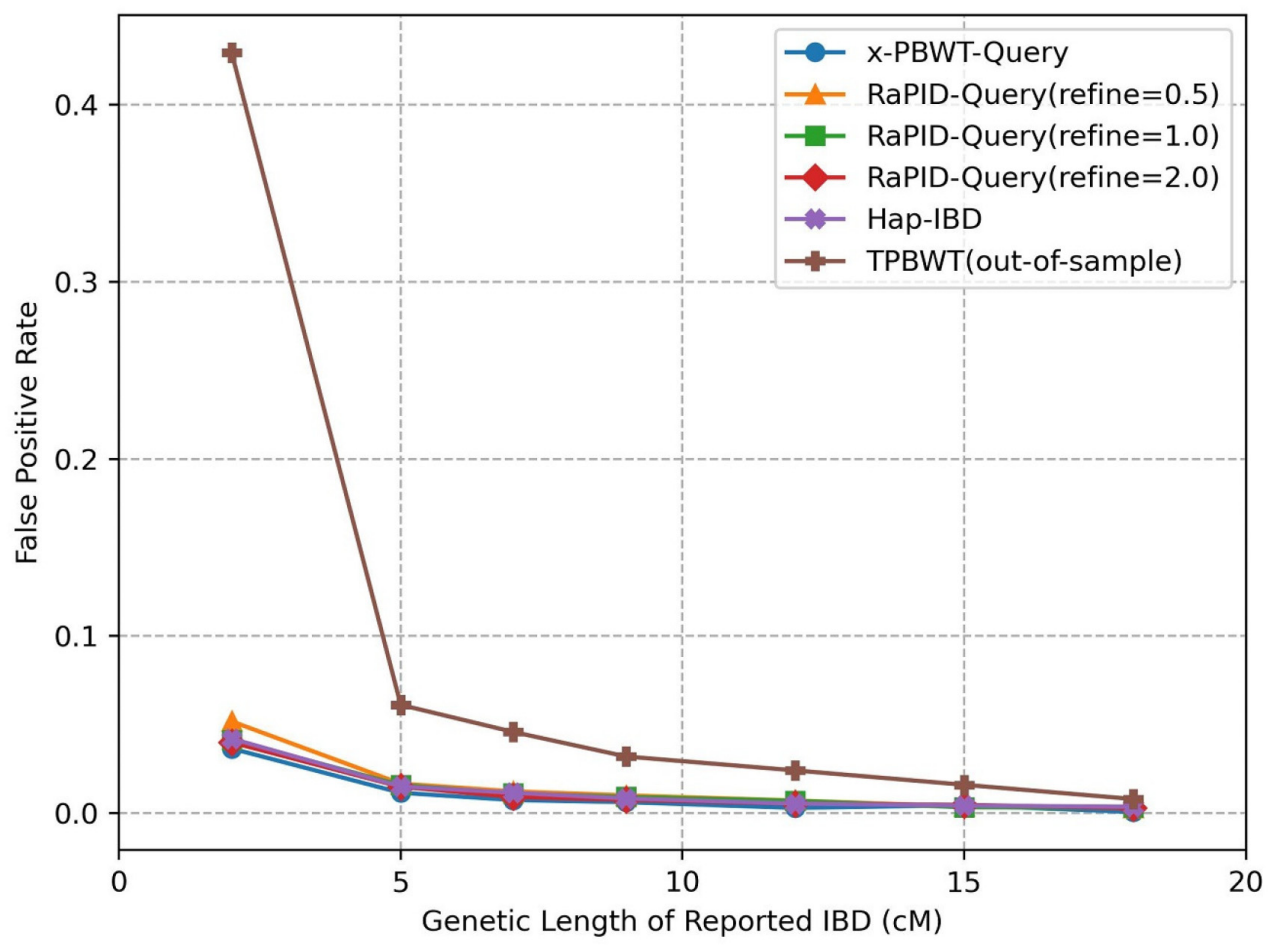
False positive rates. Reported IBD segments with length ≥2 cM were assigned into bins of 2–5, 5–7, 7–9, 9–12, 12–15, 15–18, and ≥18 cM according to their genetic length. The false positive rate is the proportion of reported IBD segments in a bin that are not covered by any true IBD segment ≥1.5 cM.

Figure 5 shows the false negative rates of five one-versus-all IBD detection methods: x-PBWT-Query, RaPID-Query(refine = 0.5), RaPID-Query(refine = 1.0), RaPID-Query(refine = 2.0), TPBWT(out-of-sample), and Hap-IBD. Overall, as the genetic length of true IBD increases, the false negative rate decreases. The x-PBWT-Query method has the worst false negative rate as it does not allow mismatches. It misses some of the IBD segments which are not exact matches and caused by genotyping errors, mutations, or gene conversions. All other methods have better false negative rates as they allow mismatches during the IBD calling. For RaPID-Query methods, the smaller the refinement parameter value is, the better the false negative rate the method has. In this example, TPBWT(out-of-sample) has the smallest false negative rates among all methods; however, by aggressively tolerating the errors, its false positive rates are the highest among all methods (see Fig. 6). RaPID-Query(refine = 0.5) has the second smallest false negative rate. The false negative rate of Hap-IBD is between that of RaPID-Query(refine = 1.0) and RaPID-Query(refine = 2.0) for all the cases, indicating the false negative rate of RaPID-Query is competitive to the state-of-the-art all-versus-all IBD detection method. If the cutoff genetic length of IBD is 7 cM or above, all RaPID-Query methods showing in Fig. 5 has very small false negative rate. This shows RaPID-Query is outstanding from the power of IBD segment detection perspective when performing genealogical searches.

The same experiment was conducted on a simulated SNP-array dataset. Supplementary Fig. S3a shows that RaPID-Query has similar false negative rates as on WGS datasets. Though false negative rates on WGS dataset are lower than ones in SNP-array dataset in general, the difference of false negative rates between them is within 0.03 for most IBD bins, except for IBDs in 2 cM bin. For IBD 2 cM bin, false negative rate on WGS dataset is 0.0884, and one on SNP-array dataset is 0.1804.

#### 3.2.2 False positive rate

The measurement of false positive rate is defined as the average proportion of reported IBD segments not being covered by true IBD segments, i.e. the sum of proportions of reported IBD segments not overlapped with true IBD segments over the number of reported IBD segments: where *IR* is reported IBD set, *ir* is reported IBD, *sr* is site of reported IBD, *IT* is true IBD set, *it* is true IBD, and *si* is the sampled individual ID.


(2) $$\begin{array}{c} \frac{1}{\left|IR\right|}\sum_{ir\in IR}\frac{1}{\left|ir\right|}\sum_{sr\in ir}\left[sr\notin \{it\in IT\},\;\text{where}\;ir.si=it.si\right], \end{array}$$

Figure 6 show the false positive rates of five one-versus-all IBD detection methods: x-PBWT-Query, RaPID-Query(refine = 0.5), RaPID-Query(refine = 1.0), RaPID-Query(refine = 2.0), TPBWT(out-of-sample), and Hap-IBD. Overall, as the genetic length of reported IBD increases, the false positive rate decreases. Though, beside TPBWT(out-of-sample), the false positive rates of all other five methods are close in almost all cases (i.e. the rate difference is only about 1%), there is difference among the methods. The x-PBWT-Query method has the smallest false positive rate as it does not allow mismatches. Other methods may detect inexact match IBD segments which could be false positive ones. For RaPID-Query methods, the larger the refinement parameter value is, the smaller the false positive rate the method has. In this example, RaPID-Query(refine = 2.0) has the smallest false positive rate as it detects the smallest number of exact match IBDs, which prevents over-extending the candidate IBDs resulted from the random projection runs. TPBWT(out-of-sample) has the largest false positive rates among all methods. The false positive rate of Hap-IBD is similar to that of RaPID-Query, which indicates the false positive rate of RaPID-Query is competitive to the state-of-the-art all-versus-all IBD detection method. If the cutoff genetic length of IBD is 7 cM or above, all RaPID-Query methods showing in Fig. 6 has very small false positive rate. This shows RaPID-Query is outstanding from the accuracy of IBD segment detection perspective when performing genealogical searches.

The same experiment was conducted on a simulated SNP-array dataset. Supplementary Fig. S3b shows that RaPID-Query has similar false positive rates as on WGS datasets. Though false positive rates on WGS dataset are lower than ones in SNP-array dataset in general, the difference of false positive rates between them is within 0.01 for most IBD bins, except for IBDs in 2 cM bin. For IBD 2 cM bin, false positive rate on WGS dataset is 0.0518, and one on SNP-array dataset is 0.0771.

### 3.3 IBD length discrepancy and impact of genotyping error

In addition to measurement of false negative rate and false positive rate, genetic length discrepancy of detecting IBDs were conducted on the same dataset. Genetic length discrepancy is calculated as the root mean square of length discrepancy between reported IBDs and true IBDs. The result shows RaPID-Query is capable of calling high-quality IBDs, as it had the smallest average genetic length discrepancies among all methods. Supplementary Fig. S4 shows genetic length discrepancies of methods, and the average one of RaPID-Query(refine = 0.5) was 2.99 across all bins.

To investigate the impact of genotyping error, a set of experiments were run on the simulated dataset with different level of genotyping errors added. In general, RaPID-Query has strong ability to tolerate genotyping error, as the result shows that, regardless of genotyping error rates, it was the only method had small false negative rates and false positive rates at the same time. Supplementary Fig. S5 shows the impact of different levels of genotyping error on false negative rate and false positive rate. RaPID-Query had no impact on false positive rate and a slight impact on false negative rate when genotyping error rate increased.

### 3.4 Runtime

The runtime of RaPID-Query was tested with different sets of refinement parameters (same used in correctness experiment) with x-PBWT-Query on the UK biobank SNP-array chromosome 20 dataset, containing 974 818 haplotypes with 17 197 sites (Bycroft et al. 2018). The GRCh37 human genome assembly from HapMap Phase II project (The International HapMap Consortium 2007) were used to determine the length of IBDs detected from each autosomal chromosome. For each query method, 400 haplotype queries were run with 7 cM and 700 sites as the cutoff IBD segment length. The reported runtime is the average runtime of a query against a panel, excluding the pre-process time of building PBWT panels. Due to the potential license conflict, TPBWT(out-of-sample) method is not able to run against UK biobank dataset. TPBWT(out-of-sample) was run on simulated WGS dataset and the runtime comparison with RaPID-Query is in Supplementary Fig. S6. RaPID-Query is faster for individual query, but TPBWT(out-of-sample) is faster when the batch has a large number of queries in it.

Table 1 shows the average runtime of one query against a large panel for RaPID-Query with various refinement values: 0.5, 1.0, 2.0, and x-PBWT-Query. x-PBWT-Query is the fastest method among all, as it is not involved in any multiple runs and merges as RaPID-Query does. For RaPID-Query method, the value of the refinement parameter is inversely proportional to the average query time. This is expected as RaPID-Query needs more time to refine the detected IBD segments as the required refinement precision increases. Even so, the RaPID-Query(refine = 0.5) method whose refinement precision is up to 0.5 cM is fast: it takes 839.63 ms on average to complete a query and write IBDs to a file.

**Table 1.** Computation times in milliseconds of 400 queries against UK biobank chromosome 20 dataset with minimum 7 cM IBD segment length and minimum 700 markers.

Method	Average CPU time (ms)	Standard deviation	Average wall time (ms)	Standard deviation
RaPID-Query (refine = 0.5)	839.63	166.26	864.47	153.69
RaPID-Query (refine = 1.0)	211.73	64.77	219.46	57.09
RaPID-Query (refine = 2.0)	39.58	20.06	48.61	24.05
x-PBWT-Query	11.80	8.02	18.32	10.10

### 3.5 RaPID-Query for genealogical analysis

To show the detected IBD segments from RaPID-Query are robust for genealogical search, a similar analysis of sum-of-IBD-length-based relatedness degree separation (Naseri et al. 2019a) was conducted on UK biobank and simulated datasets. For UK biobank dataset, the queries contain 200 randomly-selected individuals having at least third degree of relatedness with each other, and 1000 randomly-selected unrelated individuals (excluding those 200 individuals having at least third degree of relatedness with each other). For the simulated dataset, the queries are 200-randomly-selected individuals. The genealogical search minimum cutoff length of an IBD calling for both datasets is 7 cM and 700 markers, as it is the conventional minimum match threshold values currently used by major DTC companies such as 23andMe (https://customercare.23andme.com/hc/en-us/articles/212170958-DNA-Relatives-Detecting-Relatives-and-Predicting-Relationships). RaPID-Query(refine = 2.0) was used since from previous experiments, it was the fastest method with comparable false negative and false positive rates among the methods.

**Figure 7.**
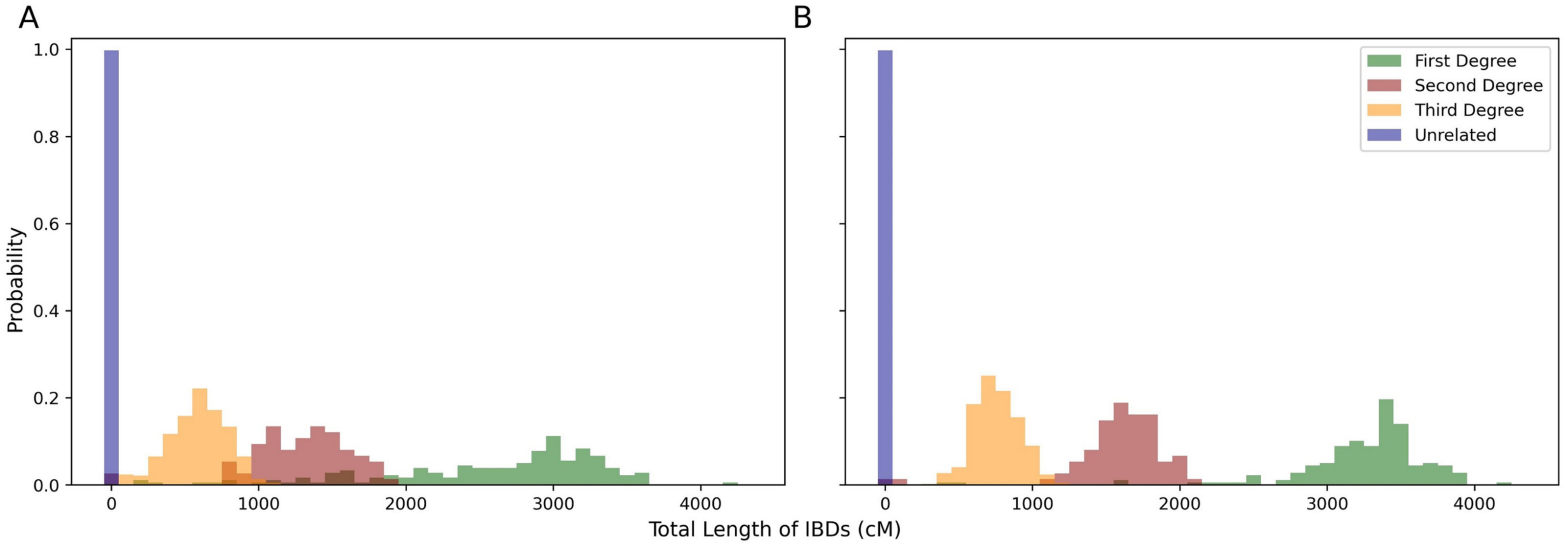
Probability distributions of sum of length of IBDs on UK Biobank dataset. (A) x-PBWT-Query. (B) RaPID-Query (refine = 2.0).

Figure 7 shows the probability distributions of total length of IBDs between individual pairs on UK biobank dataset. The probability distributions of four degrees (first-degree, second-degree, third-degree, and unrelated) in the results from x-PBWT-Query method (Fig. 7A) and from RaPID-Query(refine = 2.0) method (Fig. 7B) are distinguishable. The probability distributions resulted from x-PBWT-Query method have smaller means and larger standard deviations than those resulted from RaPID-Query method (See Supplementary Table S2). This tells that RaPID-Query identifies more robust IBDs than that of x-PBWT-Query, as RaPID-Query allows mismatched sites, which makes the reported IBDs more close to the true IBDs. On the other hand, with no support on tolerating mismatched sites, x-PBWT-Query misses many IBD segments which flattens the probability distributions of total length of IBDs between individual pairs and makes them all shifted toward right in *x* axis.

To quantify the relatedness degree separation result, the area under the receiver operating characteristic (ROC) curve (AUC) values between each distribution pairs were calculated. The AUC values of sum of length of IBDs on UK biobank dataset are shown in Table 2 (the ROC curves are in Supplementary Fig. S7). RaPID-Query’s AUC value between first- and second-degree relationships is 98.27%, and the AUC values between second- and third-degree relationships and third-degree and unrelated relationships are 97.28% and 100.00%, respectively. This indicates that IBD segments identified by RaPID-Query is capable of being used to infer up-to-third-degree familial relatedness between any individual pair in large real-world biobank. In addition, it is observed that all AUC values calculated from the sum of length of IBDs using RaPID-Query are better than those using x-PBWT-Query, which means RaPID-Query method performs better relatedness degree separation than x-PBWT-Query method does.

**Table 2.** x-PBWT-Query versus RaPID-Query(refine = 2.0): Area Under Curve (%) of Sum of Length of IBDs on UK Biobank Dataset.

Method	First degree versus second degree	Second degree versus third degree	Third degree versus unrelated
x-PBWT-Query	92.29	96.13	99.83
RaPID-Query(refine = 2.0)	98.27	97.28	100.00

The high-quality IBD segments from RaPID-Query is able to separate the fourth-degree distribution from others well in simulated dataset. Supplementary Fig. S8 shows the probability distributions of total length of IBDs between individual pairs on simulated dataset. The probability distributions of five degrees (first-degree, second-degree, third-degree, fourth-degree, and unrelated) in the results from x-PBWT-Query (Supplementary Fig. S8A) and from RaPID-Query (Supplementary Fig. S8B) are distinguishable.

The fourth-degree distribution is differentiated using the sum of length of IBDs from RaPID-Query result from other distributions. Similar to the test performed on UK biobank dataset, the AUC values in Supplementary Table S3 (the ROC curves are in Supplementary Fig. S9) between each distributions were calculated, in order to quantify the degree relatedness separation. For RaPID-Query method, the AUC value between third- and fourth-degree relationships is 98.42%, and 99.69% between fourth-degree and unrelated relationships. This indicates that RaPID-Query also has the ability to differentiate up-to-fourth-degree relationships, thanks to the output high-quality IBD segments having allowed mismatched markers and refined boundaries. The AUC values calculated from the result using x-PBWT-Query method is able to separate fourth-degree from third-degree and unrelated relationships but having lower AUC values than those calculated from RaPID-Query method. Also, the probability distributions resulted from x-PBWT-Query method have relatively smaller means than those resulted from RaPID-Query method (See Supplementary Table S4). This means it is more confident to utilize the IBD segments identified from RaPID-Query than that from x-PBWT-Query for degree of relationship separations.

From the result, it is also noticed that RaPID-Query is very fast and memory-efficient for querying autosomal chromosomes of the entire UK biobank dataset. Excluding the panel pre-processing time, the average CPU time of an individual haplotype query of RaPID-Query method is 2.76 s with 1.04 standard deviation. The average wall time is 3.05 s with 1.17 standard deviation. Naseri et al.’s PBWT-Query and L-PBWT-Query methods with 700 SNPs takes 20 and 6 s on average per query for the UK biobank dataset (excluding the panel computing and loading time) (Naseri et al. 2019a). The highest peak of memory size RaPID-Query method used to hold the pre-processed panels in memory is 1.2 terabytes, as for the PBWT-Query and L-PBWT-Query it is 2.4 and 4.7 terabytes, respectively (Naseri et al. 2019a). This shows RaPID-Query method outperforms PBWT-Query and L-PBWT-Query from both the time and space perspectives. RaPID-Query has the potential power of being scalable to population-scale cohorts with feasible query time and memory usage.

## 4 Discussion

RaPID-Query has the ability of detecting high-quality IBD segments efficiently for a given individual against a population panel. It is shown that both power and accuracy rates of RaPID-Query are comparable to the state-of-the-art IBD detection methods. The query search computation time is extraordinary compared to the previous methods, as one haplotype query takes around 3 seconds to identify IBD segments in 22 autosomal chromosomes of entire UK biobank dataset, using the conventional genealogical search threshold.

The inferred IBD segments from RaPID-Query are available for further downstream analysis, such as relationship inference. It is shown that the IBD segments are high-grade and the sum of length of those IBDs is able to categorize at least 97% individual pairs by the relationship up to 4th degree. There is a potential that the quality relationship inference for higher degrees is reachable, if the inference method utilize not only the sum of length of IBD segments, but the combination of the number, the lengths, or the locations of IBD segments, or even demographic data as Erlich et al. used in their long-range familial search pipeline (Erlich et al. 2018). The multi-step IBD-segment-based relationship inference methods, for instances, CREST (Qiao et al. 2021), DRUID (Ramstetter et al. 2018), ERSA (Li et al. 2014), IBDkin (Zhou et al. 2020b), PONDEROSA (Williams et al. 2020), may benefit from RaPID-Query if they perform their analysis based on high-quality IBD segments produced by RaPID-Query. If multiple individuals are needed for pedigree constructions or machine learning models during the inference, it is easy to run RaPID-Query on a parallel querying basis as each query is independent.

## Supplementary Material

btad312_Supplementary_Data
