# Supplementary material for: RaPID-Query for fast identity by descent search and genealogical analysis
Source: Bioinformatics. 2023 May 11;39(6):btad312. doi: 10.1093/bioinformatics/btad312 (PMC10244210; doi:10.1093/bioinformatics/btad312)
Supplement: btad312_Supplementary_Data [file btad312_supplementary_data.pdf]

Supplementary Material for "RaPID-Query for fast identity by descent search and genealogical analysis"

Yuan Wei[1], Ardalan Naseri[2], Degui Zhi[2], and Shaojie Zhang[1]

[1]Department of Computer Science
University of Central Florida, Orlando, FL, USA

[2]School of Biomedical Informatics
University of Texas Health Science Center at Houston, Houston, TX, USA

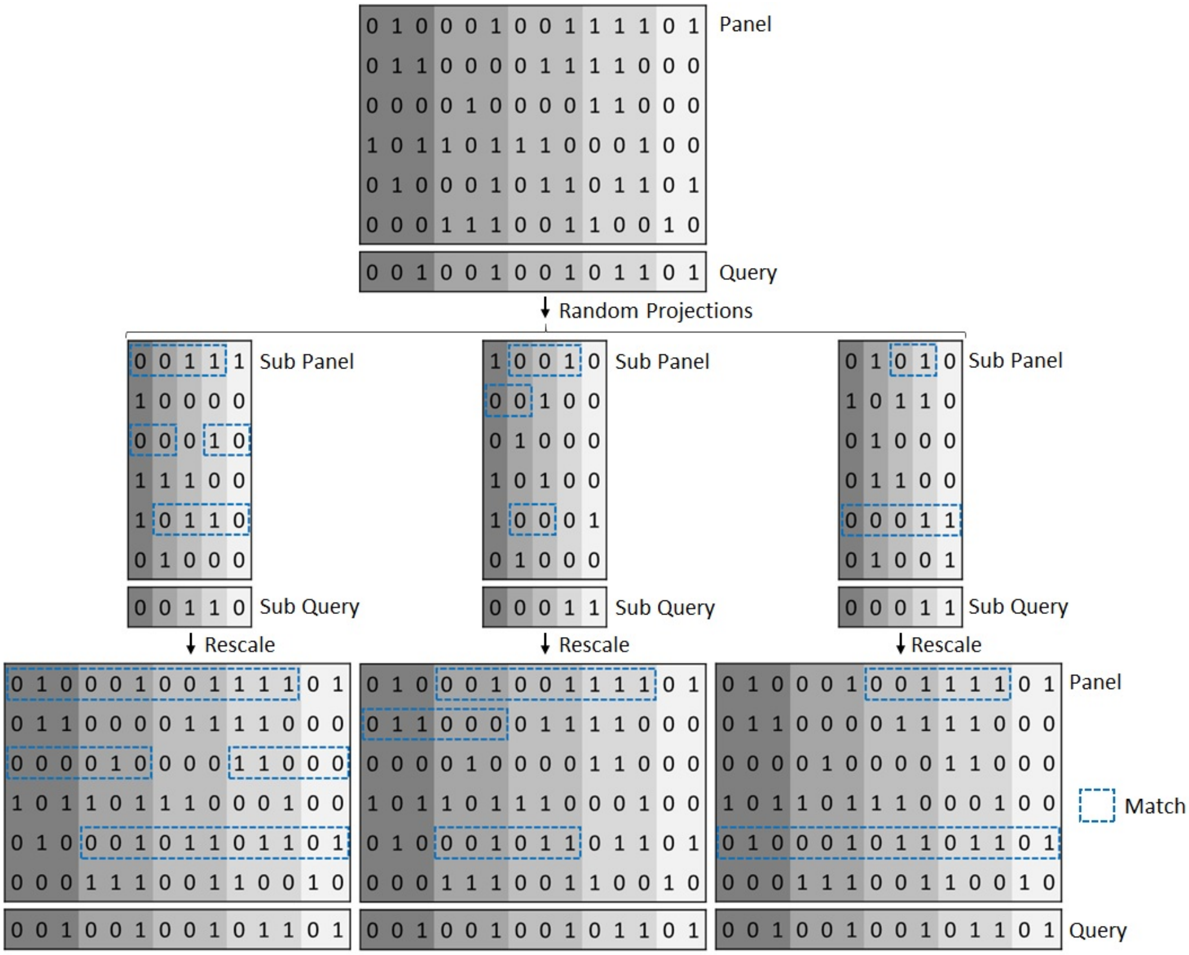

Figure S1: RaPID-Query Random Projection Example (n=14, w=3, r=3, L=6). The shading color indicates the window. One site is randomly sampled (weighted on the minor allele frequency) for each window to form $r = 3$ sub panels having $\lceil \frac{n}{w} \rceil = \lceil \frac{14}{3} \rceil = 5$ sites. The matches with at least $\frac{L}{w} = \frac{6}{3} = 2$ length in sub panels are identified by using x-PBWT-Query algorithm, and then rescaled to the original resolution.

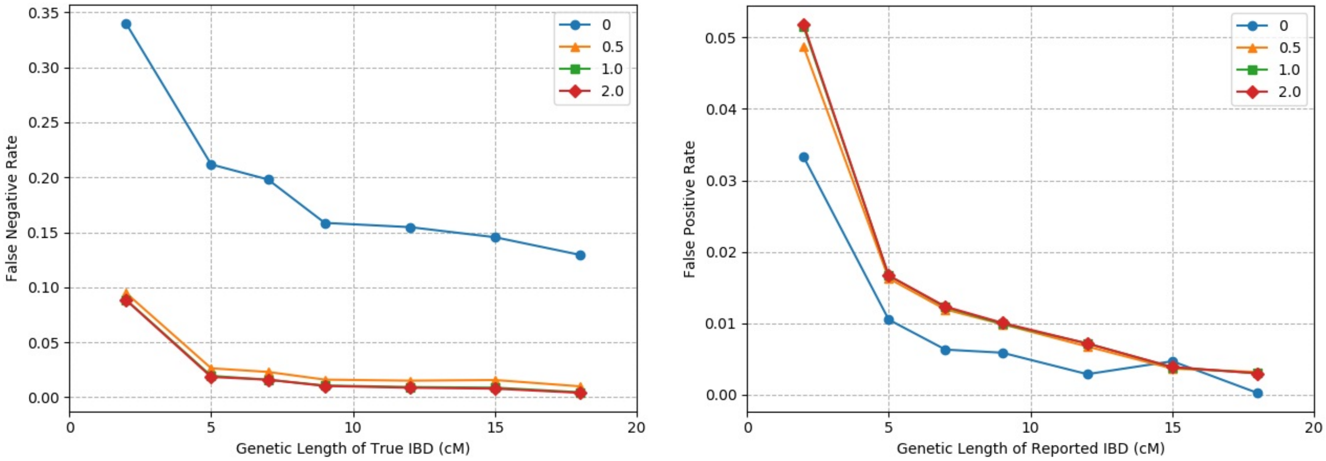

(a) False Negative Rates

(b) False Positive Rates

Figure S2: False Negative Rates and False Positive Rates with Different RaPID-Query's Gap Parameter Values. RaPID-Query's gap parameter refines IBD segments by stitching candidate IBDs together if IBD is broken by window locations. Four different gap parameter values were tested on the simulated chromosome 20 Whole Genome Sequencing (WGS) dataset: 0, 0.5, 1.0, and 2.0. IBD segment target length is 2 cM and 200 sites. The result shows gap parameter helps decreasing false negative rate while maintaining similar false positive rate: for gap parameter value 0.5, the false negative rate decreases at a range around 12.0% to 24.5%, while the false positive rate increases only at a range around 0.3% to 1.5%; for gap parameter value 1.0 and 2.0, the false negative rate decreases further but the difference is small from using value 0.5 (i.e. $< 0.6\%$); the false positive rate increases a small difference from using value 0.5 (i.e. $< 0.3\%$).

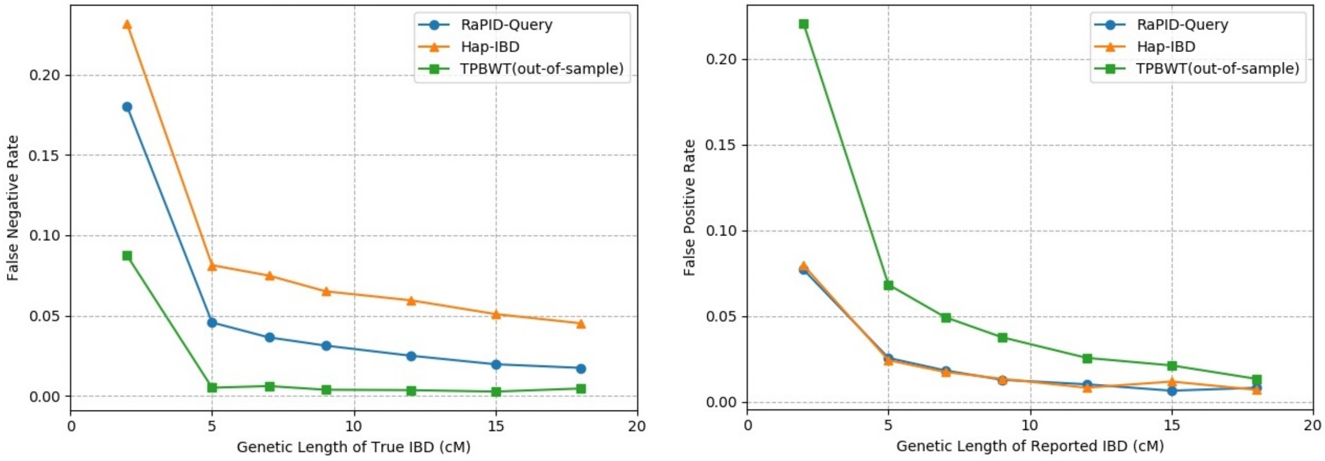

(a) False Negative Rates

(b) False Positive Rates

Figure S3: False Negative Rates and False Positive Rates on Simulated SNP-array Dataset. A chromosome 20 SNP-array dataset was generated by down-sampling the simulated WGS dataset. In particular, the markers were filtered as close to ones in UK biobank chromosome 20 dataset. It ended up with 17,925 sites. Genotyping error with rate 0.13% and phasing error with rate 0.42% were added to the SNP-array dataset. IBD segment target length is 2 cM and 100 sites. Since the number of sites in SNP-array dataset is smaller than that in WGS dataset, RaPID-Query used small cutoff site parameter (i.e. 2 cM and 100 sites for low-resolution panels, 1 cM and 50 sites for full-resolution panel). The result shows RaPID-Query has small false positive rate as Hap-IBD does, and better false negative rate than Hap-IBD; TPBWT(out-of-sample) has the smallest false negative rate and largest false positive rate among the methods. This conclusion aligns with the result of WGS dataset.

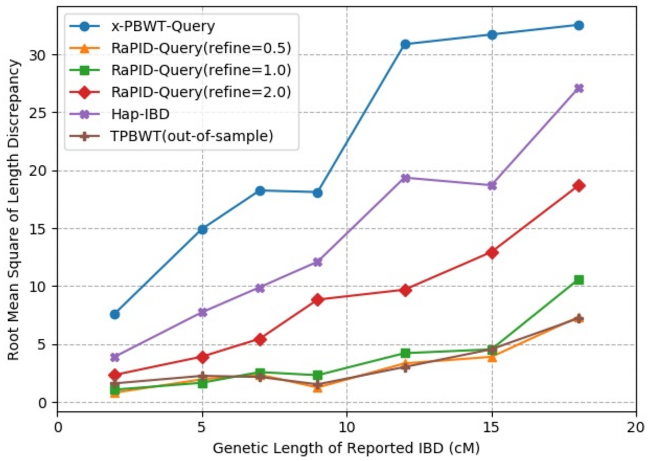

Figure S4: Genetic Length Discrepancies of Detecting IBD Segments with 2 cM and 200 sites Target Length on Simulated WGS Dataset. The genetic length discrepancy is calculated as the root mean square of length discrepancy between reported IBDs and true IBDs. Overall, RaPID-Query(refine=0.5) and TPBWT(out-of-sample) have small genetic length discrepancies (2.99 for RaPID-Query(refine=0.5) and 3.19 for TPBWT(out-of-sample) on average across all bins), compared to other methods.

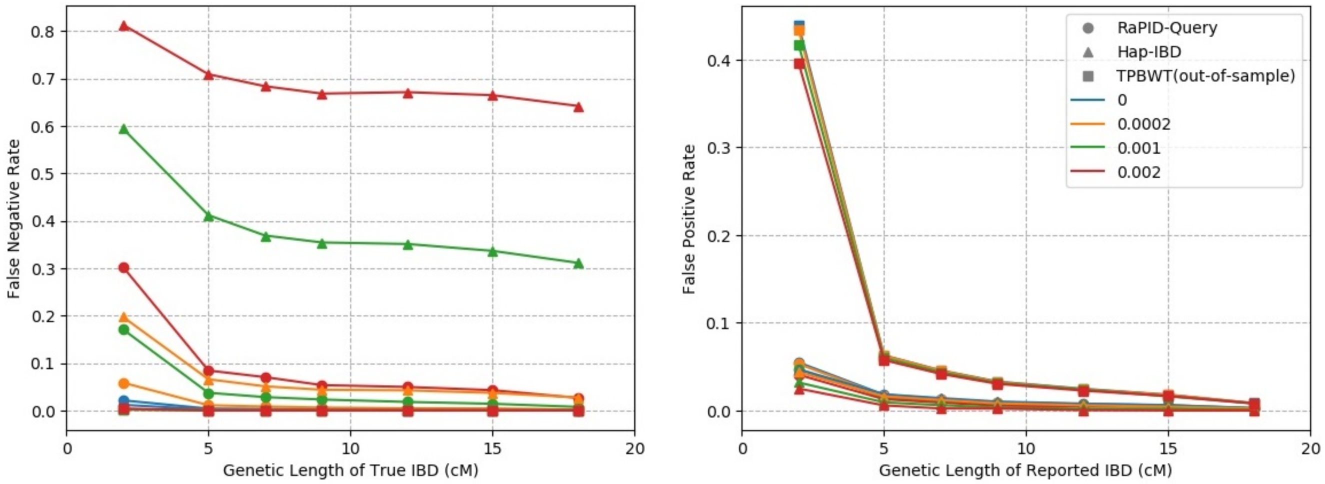

(a) False Negative Rates

(b) False Positive Rates

Figure S5: False Negative Rates and False Positive Rates with Different Levels of Genotyping Error. Four different genotyping error rates were added to the simulated chromosome 20 WGS dataset: 0%, 0.02%, 0.1%, and 0.2%. IBD segment target length is 2 cM and 200 sites. Overall, all methods have consistent false positive rates with different levels of genotyping error. For false negative rates, TPBWT(out-of-sample) does not have much impact, RaPID-Query has slight impact, and Hap-IBD has large impact. RaPID-Query is the only method able to achieve small false negative rate and false positive rate at the same time on panels with different levels of genotyping errors. When genotyping error rate increases, false negative rate slightly increases, and false positive rate is consistently small. TPBWT(out-of-sample) has the smallest false negative rates but the largest false positive rates for all bins among all methods. Hap-IBD is able to maintain consistently small false positive rates when genotyping error rate increases; however, its false negative rate is greatly impacted by the genotyping error rate.

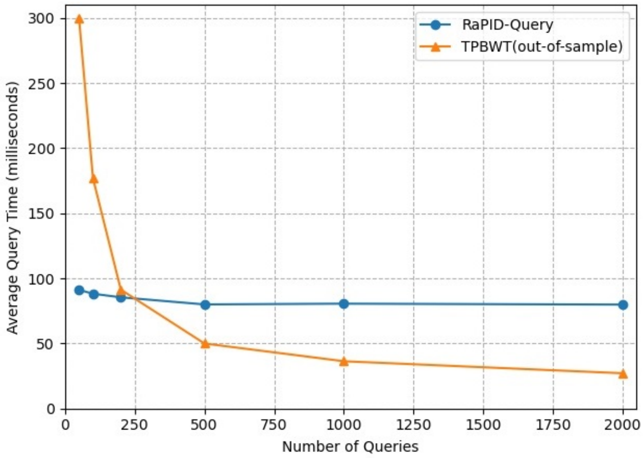

Figure S6: Average Central Processing Unit (CPU) Time for Detecting IBD Segments per Query. Six query batches with different number of queries (50, 100, 200, 500, 1,000, and 2,000) with 2 cM and 200 sites target length were run on simulated chromosome 20 WGS dataset. The pre-process time of the reference panel was not included as part of the actual query time (for RaPID-Query, it is to build PBWT panels for the reference panel; for TPBWT(out-of-sample), it is to compress the reference panel) since the pre-process step is independent from the queries and able to be prepared ahead of time. For TPBWT(out-of-sample) the query runtime is amortized as the time of loading and processing the pre-compressed reference panel was included. RaPID-Query has a constant average query time regardless of the number of queries, and is faster when the number of queries in the batch is 250 or less; average query time of TPBWT(out-of-sample) decreases as the number of queries increases, and is faster if the batch has more than 250 queries on the dataset. This is because RaPID-Query is a real query-based approach where each query runs in a linear fashion of the number of sites n in the panel (i.e. $O(n)$). On the other hand, TPBWT(out-of-sample) is a batch-based approach which appends queries to the panel and runs the original TPBWT, with outputting IBDs between individuals in the queries and individuals in the panel only. The time complexity is $O(n(m_p + m_q))$, where m_p is the number of individual haplotypes in the panel and m_q is the total number of individual haplotypes to be queried. Thus, the more queries it has, the faster each query runtime is on average as it scans the panel regardless of the number of queries. From theoretical perspective, RaPID-Query is faster when $nm_q < \frac{n(m_p+m_q)}{m_q}$, which is $m_q < \frac{1+\sqrt{1+4m_p}}{2}$.

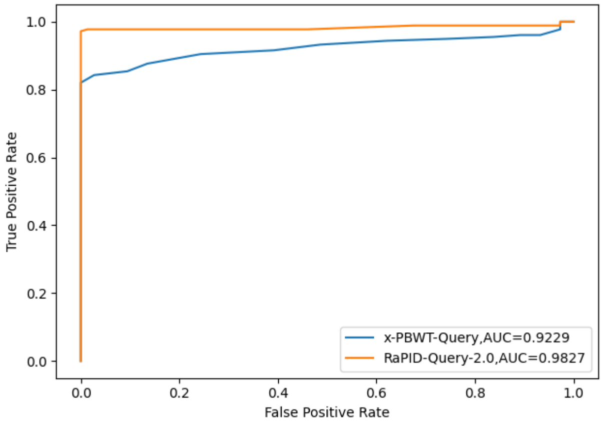

(a) 1st Degree vs 2nd Degree

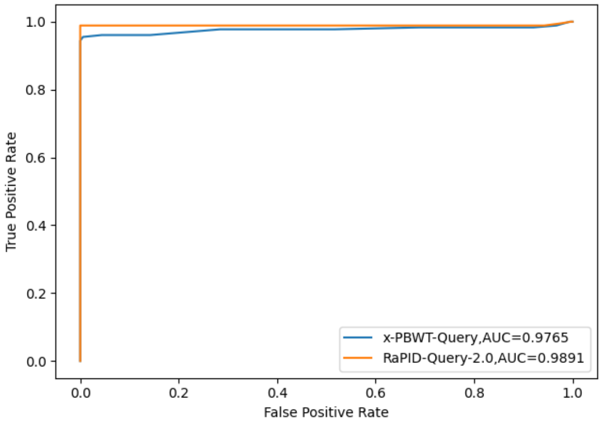

(b) 1st Degree vs 3rd Degree

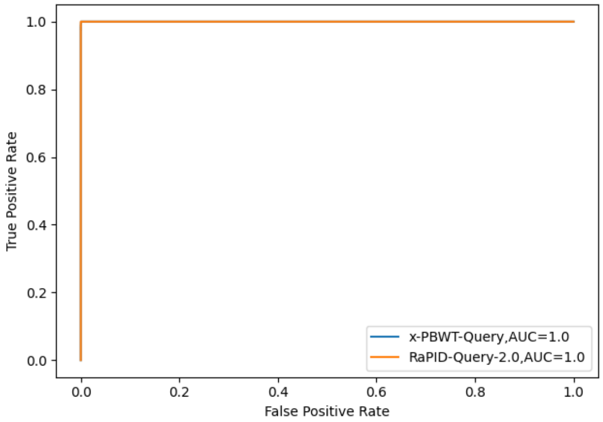

(c) 1st Degree vs Unrelated

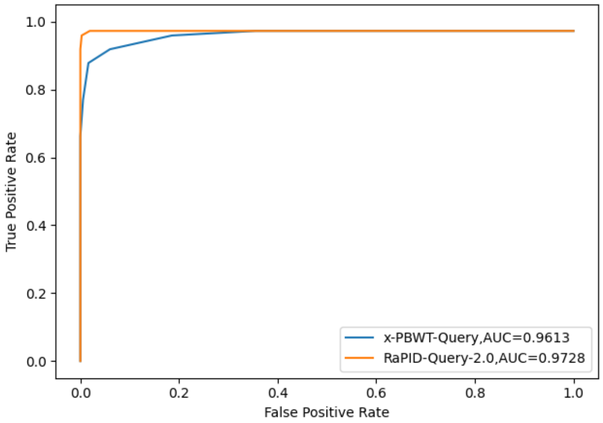

(d) 2nd Degree vs 3rd Degree

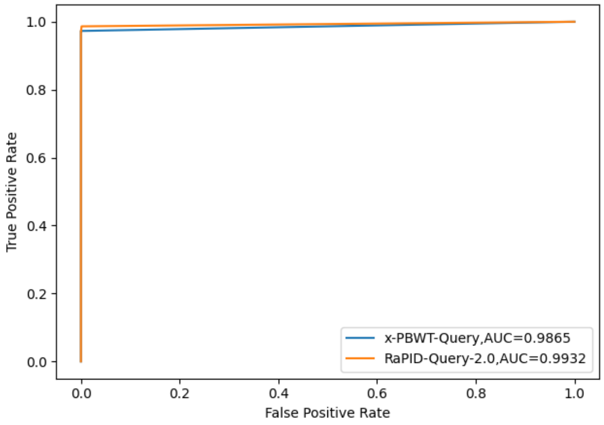

(e) 2nd Degree vs Unrelated

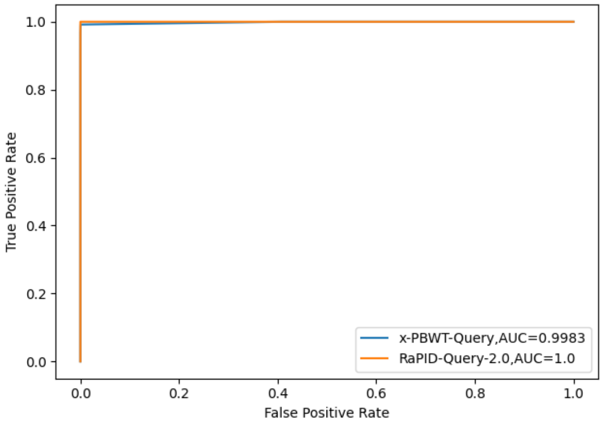

(f) 3rd Degree vs Unrelated

Figure S7: ROC Curves and AUC Values of Sum of Length of IBDs on UK Biobank Dataset.

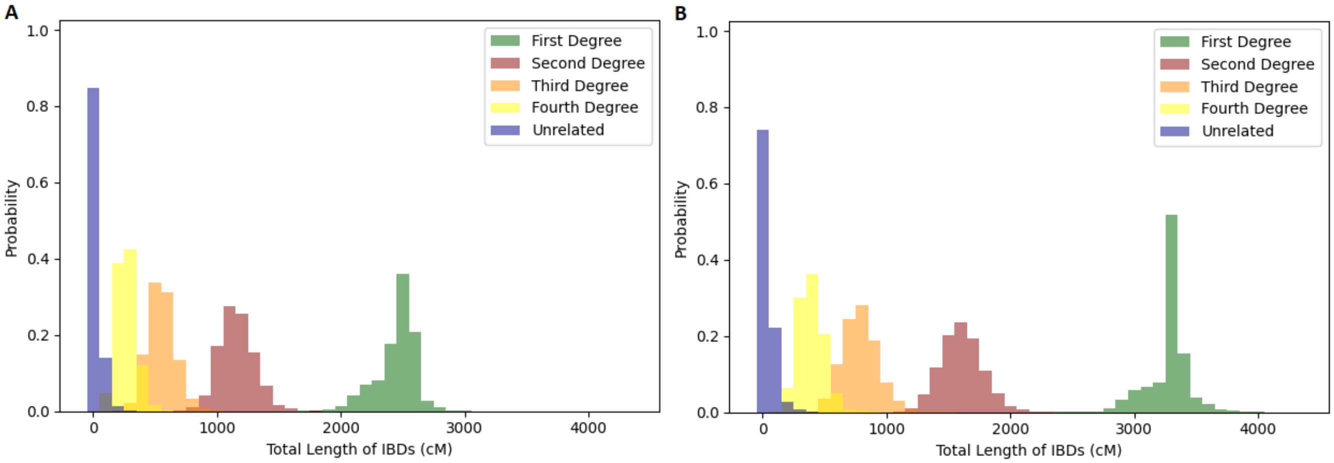

Figure S8: Probability Distributions of Sum of Length of IBDs on Simulated Dataset. (A) x-PBWT-Query. (B) RaPID-Query-2.0.

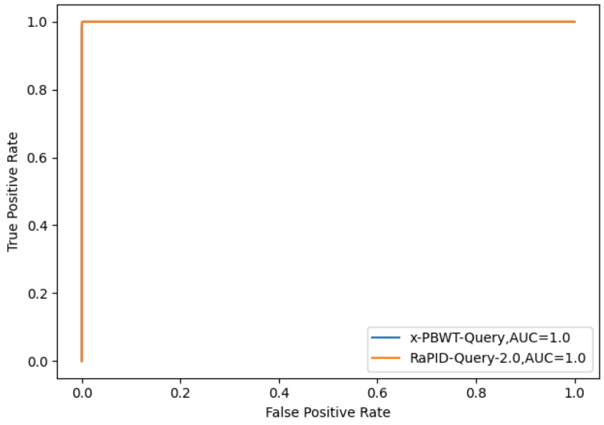

(a) 1st Degree vs 2nd Degree

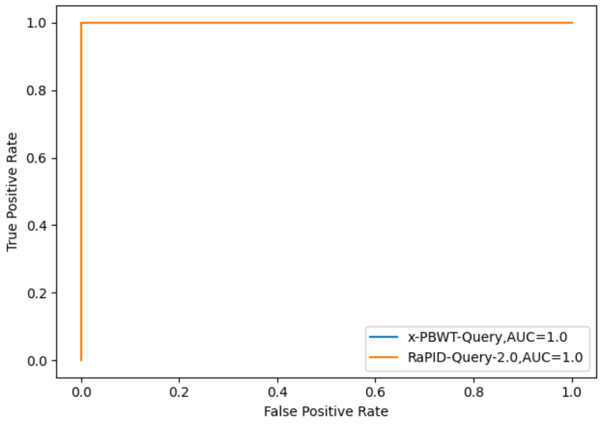

(b) 1st Degree vs 3rd Degree

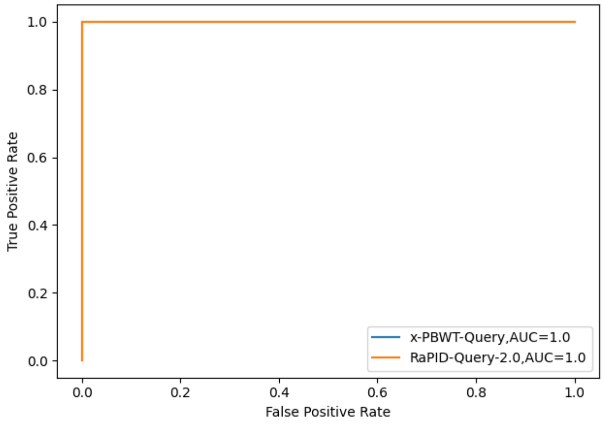

(c) 1st Degree vs 4th Degree

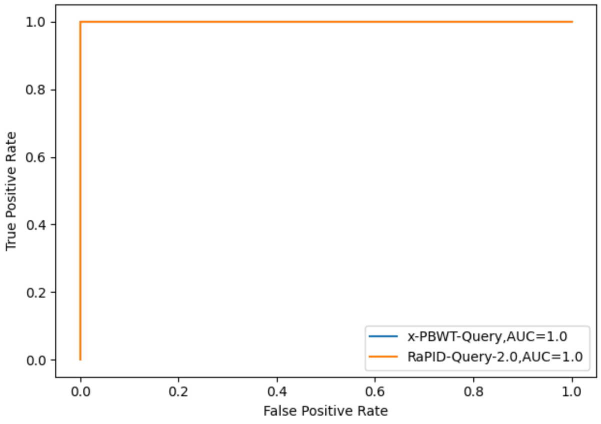

(d) 1st Degree vs Unrelated

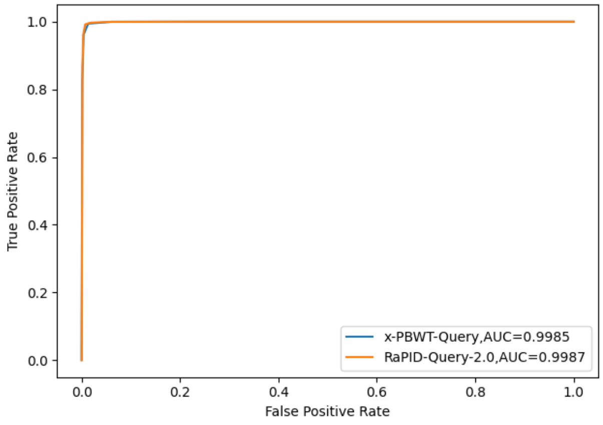

(e) 2nd Degree vs 3rd Degree

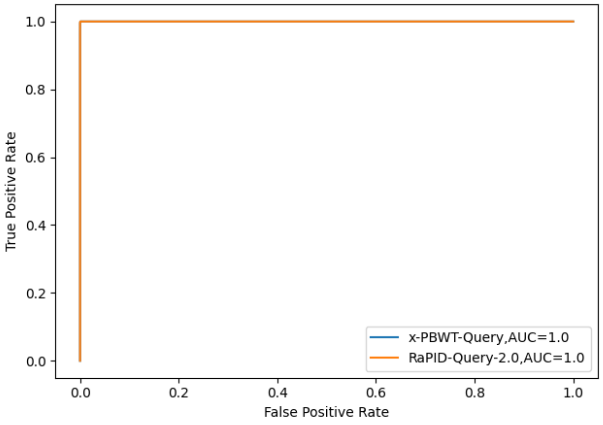

(f) 2nd Degree vs 4th Degree

Figure S9: ROC Curves and AUC Values of Sum of Length of IBDs on Simulated Dataset.

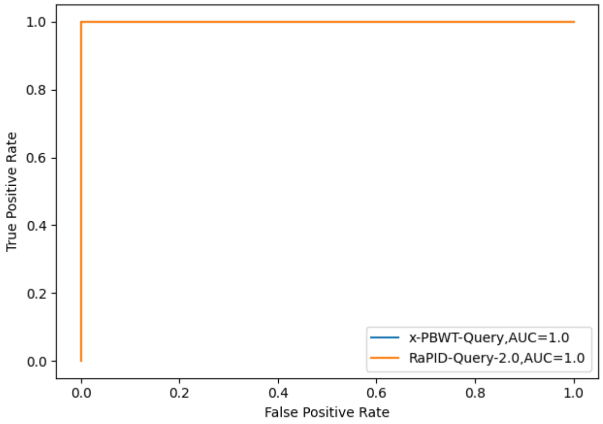

(g) 2nd Degree vs Unrelated

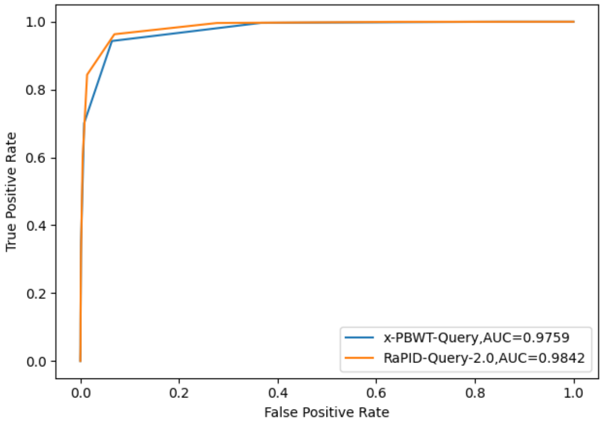

(h) 3rd Degree vs 4th Degree

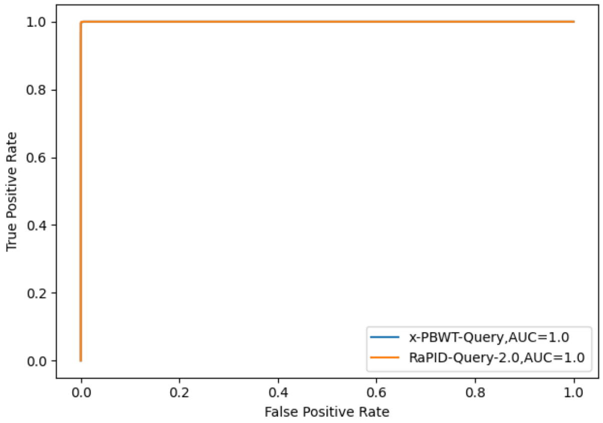

(i) 3rd Degree vs Unrelated

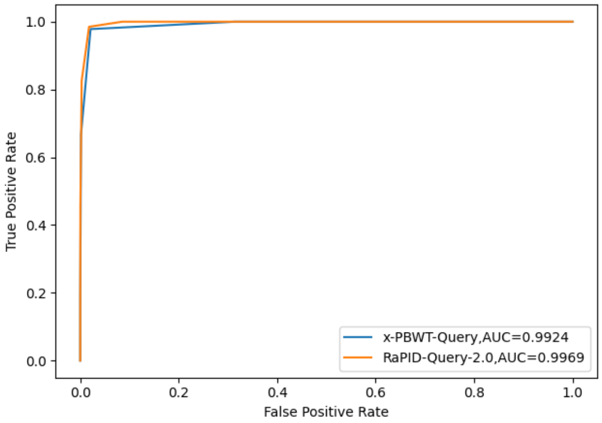

(j) 4th Degree vs Unrelated

Figure S9: ROC Curves and AUC Values of Sum of Length of IBDs on Simulated Dataset (Continued).

Method	Parameters
x-PBWT-Query	-d 2.0 -lm 100
RaPID-Query(refine=0.5)	-w 13 -r 5 -c 1 -d 2.0 -lm 200 -dh 0.5 -lmh 100 -dg 2.0
RaPID-Query(refine=1.0)	-w 13 -r 5 -c 1 -d 2.0 -lm 200 -dh 1.0 -lmh 100 -dg 2.0
RaPID-Query(refine=2.0)	-w 13 -r 5 -c 1 -d 2.0 -lm 200 -dh 2.0 -lmh 100 -dg 2.0
Hap-IBD(v1.0)	min-seed=2.0 min-extend=1.0 min-output=2.0 max-gap=1000 min-markers=100 min-mac=2
TPBWT(out-of-sample)	L_m=200 L_f=2.0

Table S1: Parameters used for performance analysis of simulated chromosome 20 Whole Genome Sequencing (WGS) dataset with minimum 2 cM IBD segment length and minimum 200 markers.

**Degree Distribution**	**x-PBWT-Query**		**RaPID-Query-2.0**	
	Mean	Standard Deviation	Mean	Standard Deviation
1st Degree	2594.94	754.78	3269.66	544.51
2nd Degree	1287.84	347.56	1591.89	330.75
3rd Degree	591.80	195.69	756.28	158.20
Unrelated	0.22	5.78	0.23	6.17

Table S2: Probability Distributions of Sum of Length of IBDs Parameters on UK Biobank Dataset.

**Method**	**1st Degree versus 2nd Degree**	**2nd Degree versus 3rd Degree**	**3rd Degree versus 4th Degree**	**4th Degree versus Unrelated**
x-PBWT-Query	100.00	99.85	97.59	99.24
RaPID-Query-2.0	100.00	99.87	98.42	99.69

Table S3: x-PBWT-Query versus RaPID-Query-2.0: Area Under Curve (%) of Sum of Length of IBDs on Simulated Dataset.

Degree Distribution	x-PBWT-Query		RaPID-Query-2.0	
	Mean	Standard Deviation	Mean	Standard Deviation
1st Degree	2448.88	163.15	3276.03	167.17
2nd Degree	1158.86	145.00	1605.77	178.79
3rd Degree	553.29	115.86	788.05	148.96
4th Degree	267.64	82.88	391.27	107.71
Unrelated	16.74	41.34	30.21	56.36

Table S4: Probability Distributions of Sum of Length of IBDs Parameters on Simulated Dataset.

Algorithm S1 x-PBWT-Query

```
f_L = 0, g_L = 0, e = 0, f_e = 0, g_e = m, i = 0, h = null
for k from 0 to n − 1 do
    // update site index currently L away from k
    while i < k + 1 − L do
        i = i + 1
    // report match
    if h is not null then
        f_r = getBlockIndicator(f_L, k, \overline{z[k]}, true)
        g_r = getBlockIndicator(g_L, k, \overline{z[k]}, false)
        for j from f_r to g_r − 1 do
            if p[k][j] is in h then
                report (p[k][j], h[p[k][j]], k − 1)
                h.remove(p[k][j])
    // update search block
    f'_e = getBlockIndicator(f_e, k, z[k], true)
    g'_e = getBlockIndicator(g_e, k, z[k], false)
    if f'_e < g'_e then
        e' = e
    else
        if f'_e == 0 or f'_e == m then
            e' = k
        else
            e' = d[k][f'_e] − 1
        if (f'_e == m and z[e'] == X[p[k][m − 1]][e']) or (f'_e > 0 and f'_e < m and z[e'] == 0) then
            f'_e = f'_e − 1
            while e' ≥ 1 and z[e' − 1] == X[p[k][f'_e]][e' − 1] do
                e' = e' − 1
            while f'_e > 0 and d[k][f'_e] ≤ e' do
                f'_e = f'_e − 1
        else if (g'_e == 0 and z[e'] == X[p[k][0]][e']) or (g'_e > 0 and g'_e < m and z[e'] == 1) then
            while e' ≥ 1 and z[e' − 1] == X[p[k][g'_e]][e' − 1] do
                e' = e' − 1
            g'_e = g'_e + 1
            while g'_e < m and d[k][g'_e] ≤ e' do
                g'_e = g'_e + 1
        else
            f'_e = 0, g'_e = m, e' = k + 1
    e = e', f_e = f'_e, g_e = g'_e
```

Algorithm S1 x-PBWT-Query (Continued)

   // *update match block*
   if $f_L < g_L$ **then**
      $f'_L = getBlockIndicator(f_L, k, z[k], \textbf{true})$
      $g'_L = getBlockIndicator(g_L, k, z[k], \textbf{false})$
   else
      $f'_L = f_L$
      $g'_L = g_L$
   if $f'_L == g'_L$ **then**
      if $e == k + 1 - L$ **then**
         for j from f_e to $g_e - 1$ **do**
            $h[p[k][j]] = e$
         $f'_L = f_e, g'_L = g_e$
   if $f'_L < g'_L$ **then**
      while $d[k][f'_L] \leq k + 1 - L$ **do**
         $f'_L = f'_L - 1$
         $h[p[k][f'_L]] = i$
      while $d[k][g'_L] \leq k + 1 - L$ **do**
         $h[p[k][g'_L]] = i$
         $g'_L = g'_L + 1$
   $f_L = f'_L, g_L = g'_L$

Algorithm S2 Get Search and Match Block Indicator

function GETBLOCKINDICATOR($indicator, k, site\_value, is\_indicator\_f$)
   if $indicator == m$ **then**
      if $is\_indicator\_f$ **then**
         if $site\_value == 0$ **then**
            $indicator = u[k][0]$
         else
            $indicator = v[k][0]$
      else
         if $site\_value == 0$ **then**
            $indicator = v[k][0]$
         else
            $indicator = m$
   else
      if $site\_value == 0$ **then**
         $indicator = u[k][indicator]$
      else
         $indicator = v[k][indicator]$
   return $indicator$

Algorithm S3 Collect Detected Match

for k from 0 to $n-1$ **do**
  for each new detected match of haplotype id_{new} starting at h_{new} **do**
    // merge detected matches ending at k
    if h_{new} is not in $B_a[k][id_{new}].H$ **then**
      $B_a[k][id_{new}].H.add(h_{new})$
      $B_a[k][id_{new}].H.h_{new}.count = 1$
    for each h in $B_a[k][id_{new}].H$ **do**
      if $h > h_{new}$ **then**
        $B_a[k][id_{new}].H.h.count++$
    // merge detected matches ending at less than k
    $h_{min} = \min_{h \in B_a[k][id_{new}].H}(B_a[k][id_{new}].H.h)$
    $i = k - w$
    while $i \geq h_{min}$ **do**
      for each h in $B_a[i][id_{new}].H$ **do**
        if $h \geq h_{min}$ **then**
          $B_a[i][id_{new}]H.h.count++$
      $i = i - w$

Algorithm S4 Identify Fragmented IBD

for k from 0 to $n-1$ **do**
  for each id in $B_a[k]$ **do**
    for each h in $B_a[k][id].H$ **do**
      if $B_a[k][id].H.h.count < c$ **then**
        $B_a[k][id].H.remove(h)$
    if $B_a[k][id]$ is null **then**
      $B_a[k].remove(id)$

Algorithm S5 Consolidate Candidate IBD

for k from 0 to $n-1$ **do**
    for each id in $B_a[k]$ **do**
        // merge overlapped fragmented IBDs ending at k
        $h_{min} = \min_{h \in B_a[k][id].H}(B_a[k][id].H.h)$
        for each h in $B_a[k][id].H$ **do**
            if $B_a[k][id].H.h > h_{min}$ **then**
                $B_a[k][id].H.remove(h)$
        // merge overlapped fragmented IBDs ending at less than k
        $i = k - w$
        while $i \geq h_{min} - 1$ **do**
            if id is in $B_a[i]$ **then**
                $h_{prev} = B_a[i][id].H.h$
                $B_a[i][id].H.remove(h)$
                $B_a[i].remove(id)$
                if $h_{prev} < h_{min}$ **then**
                    $B_a[k][id].H.h = h_{prev}$
                    break
            $i = i - w$

Algorithm S6 Convert Candidate IBD Form

for k from $n-1$ to 0 **do**
    for each id in $B_a[k]$ **do**
        $B_m[id].S.push((B_a[k][id].H.h, k))$

Algorithm S7 Refine Candidate IBD

```
for each (id, [h_full, t_full]) candidate IBD from querying full panel do
    if B_m[id] is not null then
        (h_low, t_low) = B_m[id].S.top()
        while t_low < t_full do
            B_m[id].S.pop()
            (h_low, t_low) = B_m[id].S.top()
        if (h_low, t_low) has overlap with (h_full, t_full) then
            if B_m[id].R is null then
                if t_low ≤ t_full then
                    B_m[id].S.pop()
                    report (id, [max(h_low, h_full), t_low])
                else
                    B_m[id].R = (max(h_low, h_full), t_full)
            else
                (h_R, t_R) = B_m[id].R
                if t_low ≤ t_full then
                    B_m[id].S.pop()
                    if h_full − t_R ≤ g_max then
                        report (id, [h_R, t_low])
                    else
                        report (id, [h_R, t_R])
                        report (id, [h_full, t_low])
                    B_m[id].R = null
                else
                    if h_full − t_R ≤ g_max then
                        B_m[id].R = (h_R, t_Full)
                    else
                        report (id, [h_R, t_R])
                        B_m[id].R = (h_full, t_Full)
```
